# Structural and Spectroscopic Characterization of Two Nitropyridine Amino *N*-Oxide Derivatives

**DOI:** 10.3390/ijms27146216

**Published:** 2026-07-12

**Authors:** Patrycja Godlewska, Jan Janczak, Wojciech Sąsiadek, Edyta Kucharska, Paulina Ropuszyńska-Robak, Lucyna Dymińska, Radosław Lisiecki

**Affiliations:** 1 Department of Bioorganic Chemistry, Faculty of Production Engineering, Wroclaw University of Economics and Business, 118-120 Komandorska Str., 53-345 Wrocław, Poland; wojciech.sasiadek@ue.wroc.pl (W.S.); edyta.kucharska@ue.wroc.pl (E.K.); paulina.ropuszynska-robak@ue.wroc.pl (P.R.-R.); lucyna.dyminska@ue.wroc.pl (L.D.); 2 Institute of Low Temperature and Structure Research, Polish Academy of Sciences, Okólna 2, 50-422 Wrocław, Poland; j.janczak@intibs.pl (J.J.); r.lisiecki@intibs.pl (R.L.)

**Keywords:** pyridine *N*-oxides, nitropyridine derivatives, hydrogen bonding, Hirshfeld surface analysis, FT-IR, Raman, UV–Vis, photoluminescence, DFT, solid-state NMR

## Abstract

Two new nitropyridine amino *N*-oxide derivatives were synthesized and characterized: 3-*N*-methylamino-4-nitropyridine *N*-oxide (NOPCH_3_) and [(4-nitropyridine-3-yl)amino]propanoic acid *N*-oxide (NOPCOOH)1. Their molecular and crystal structures were determined by single-crystal X-ray diffraction, and the intermolecular contact patterns were evaluated using Hirshfeld surface analysis. Vibrational properties were investigated by FT-IR and Raman spectroscopy and interpreted with the support of quantum-chemical calculations (DFT). Electronic absorption was examined by UV–Vis measurements, while photoluminescence spectra and decay profiles were recorded under femtosecond excitation and interpreted conservatively in terms of relaxed excited-state emission. Solid-state ^13^C CP-MAS and ^1^H WPMLG NMR spectroscopy provided additional support for the chemical constitution of both derivatives, with ^1^H spectra interpreted conservatively because of the limitations inherent to solid-state homonuclear-decoupled measurements. A comparative analysis shows that replacing the methyl substituent (NOPCH_3_) with the carboxylic-acid side chain (NOPCOOH) modifies the hydrogen-bonding landscape and the local electronic environment of the NO_2_ group, which is reflected in the positions and intensities of selected NO_2_-related vibrational bands and in the near-UV electronic transitions. Overall, the combined structural and spectroscopic dataset provides a consistent structure–property picture for both *N*-oxide derivatives and a basis for further studies of their coordination and photophysical behavior.

## 1. Introduction

In our previous study, the hydroxy analogue 3-hydroxyamino-4-nitropyridine *N*-oxide (NOPOH) was synthesized and structurally and spectroscopically characterized [1]. The present work extends this series to two new nitropyridine amino *N*-oxide derivatives containing N-methylamino and N-(propanoic acid)amino substituents. Amino–nitro pyridine *N*-oxides belong to a broad family of heterocyclic compounds that have been reported in diverse chemical and biological contexts, including substances identified in natural sources (plants as well as marine and microbial matrices). Pyridine *N*-oxides are also widely investigated for practical applications in industry, medicine, biochemistry, and nanotechnology [2,3,4,5,6,7,8,9,10,11,12,13,14,15,16,17,18,19,20,21]. In the literature, various substituted pyridine *N*-oxides (bearing amino, nitro, methyl, chloro, and related groups) have been associated with a range of bioactivity profiles, including antibacterial, anticancer, antihypertensive, antifungal, and anti-HIV effects [22]. Beyond the applied perspective, these systems are attractive as model molecules for correlating substitution patterns with structural and spectroscopic responses.

A substantial body of work has therefore focused on the vibrational, electronic, magnetic, and structural properties of pyridine *N*-oxide derivatives, frequently supported by quantum-chemical calculations (DFT and related approaches) [23,24,25,26,27,28,29,30,31,32,33,34,35,36,37,38,39,40]. In many cases, the key factor governing the experimentally observed properties is not only the intrinsic electronic effect of substituents but also the presence of inter- and intramolecular interactions—especially hydrogen bonding. Such interactions can measurably affect phonon-related features (IR/Raman band positions and intensities) and can also influence electronic transitions by modifying local electron density and supramolecular organization in the solid state.

This aspect becomes particularly relevant when pyridine *N*-oxide derivatives act as ligands in coordination compounds containing f- and d-electron metals [41,42,43,44,45,46,47,48,49,50,51,52,53,54,55,56,57,58,59,60,61]. In lanthanide-containing systems, direct excitation of the metal ion is often inefficient, which motivates the use of organic ligands as light-harvesting “antennae” that absorb incident radiation and transfer the excitation energy to the lanthanide center, enabling characteristic emission. Pyridine-based ligands have been widely explored in this context, and numerous lanthanide complexes with pyridine-type donors have been reported [56,57,58]. Furthermore, excitation with ultrashort (femtosecond) pulses can provide additional insight into depopulation pathways and energy-transfer mechanisms by allowing time-resolved observation of ligand-centered excited states and their relaxation.

**Scheme 1:**
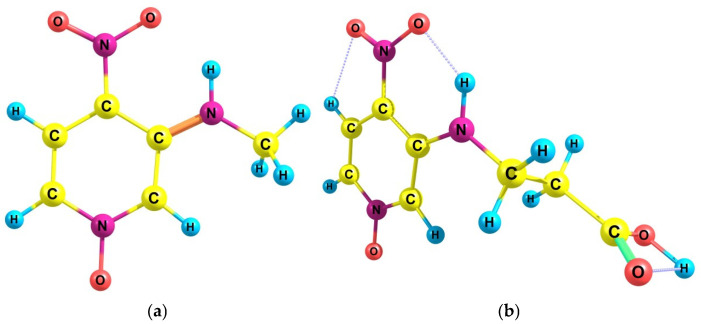
Investigated compounds: (**a**) 3-*N*-methylamino-4-nitropyridine-*N*-oxide (NOPCH_3_) and (**b**) [(4-nitropyridin-3-yl)amino]propanoic acid *N*-oxide (NOPCOOH).

In this work, we report the synthesis and comprehensive characterization of two new nitropyridine amino *N*-oxide derivatives, as shown in Scheme 1: 3-*N*-methylamino-4-nitropyridine-*N*-oxide (NOPCH_3_) and [(4-nitropyridin-3-yl)amino]propanoic acid *N*-oxide (NOPCOOH). These compounds share the same nitropyridine *N*-oxide core, while differing in the substituent attached to the amino fragment (methyl vs. carboxylic-acid side chain), providing a controlled framework to examine how the substituent modulates the hydrogen-bonding landscape and the local electronic environment of the NO_2_ group. The study combines single-crystal X-ray diffraction and Hirshfeld surface analysis with FT-IR and Raman spectroscopy, UV–Vis measurements, photoluminescence spectra and decay profiles recorded under femtosecond excitation, and solid-state ^1^H/^13^C NMR spectroscopy. The experimental data are interpreted with the support of DFT analysis, enabling consistent structure–property correlations for NOPCH_3_ and NOPCOOH and providing a basis for considering these derivatives as potential ligands in future coordination and photophysical studies.

The novelty of the present work lies in the synthesis and comparative structural–spectroscopic characterization of two previously unreported nitropyridine amino *N*-oxide derivatives that share the same nitropyridine *N*-oxide chromophore but differ in the substituent attached to the amino fragment. Previous studies of related pyridine *N*-oxide and nitropyridine derivatives have provided valuable information on the influence of substitution, hydrogen bonding, and coordination ability on their structural and spectroscopic properties. However, the present study provides a direct comparison between a compact *N*-methylamino derivative (NOPCH_3_) and a derivative bearing an additional propanoic-acid side chain (NOPCOOH). This pair of compounds enables the substituent effect to be examined in a controlled way, particularly with respect to hydrogen-bonding motifs, the local environment of the NO_2_ and *N*-oxide functionalities, and the resulting vibrational, electronic, and NMR spectral signatures. Therefore, the work extends previous studies by combining synthesis, single-crystal X-ray diffraction, Hirshfeld surface analysis, vibrational spectroscopy supported by DFT, diffuse-reflectance UV–Vis spectroscopy supported by TD-DFT, solid-state and solution-state NMR spectroscopy, and GIAO calculations into a single comparative molecular-level description of these two new derivatives.

Because of the presence of *N*-oxide donor sites and, in NOPCOOH, an additional carboxylic-acid functionality, the investigated compounds may serve as structurally characterized precursors for future coordination studies. However, in the present work, their role is limited to a comparative structural and spectroscopic analysis of two substituted nitropyridine amino *N*-oxide derivatives.

## 2. Results and Discussion

### 2.1. Structural Characterization

The effect of substituents in the pyridine ring of nitropyridine amino *N*-oxide derivatives NOPCH_3_ and NOPCOOH strongly affects their structural and optical properties. The dark-orange color is characteristic of derivative NOPCH_3_ with a methylamino group substituted in the 3-position of the pyridine ring (Scheme 1a), whereas the light-orange color is characteristic of derivative NOPCOOH with a propanoic-acid side chain substituted in the 3-position of the pyridine ring (Scheme 1b). The position of substituents in these derivatives is also reflected in their crystal structures.

#### 2.1.1. Description of the Structure of 3-*N*-Methylamino-4-Nitropyridine-*N*-Oxide (NOPCH_3_)

**Figure 1:**
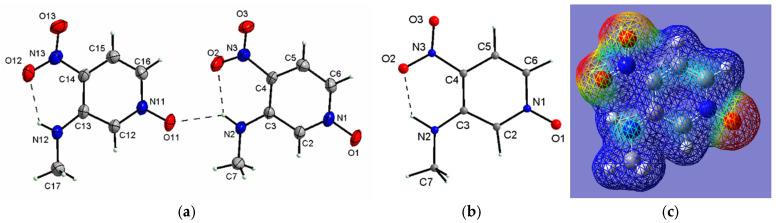
View of the X-ray structure of NOPCH_3_ (**a**), DFT-optimized molecule (**b**), and 3D MESP map of the molecule, color code of MESP maps is in the range of −0.05 (red) to 0.05 eÅ-1 (blue) (**c**). The displacement ellipsoids are shown at the 50% probability level, and H atoms are shown with arbitrary radii.

3-*N*-methylamino-4-nitropyridine-*N*-oxide (NOPCH_3_) crystallizes in a centrosymmetric triclinic space group with four molecules per unit cell. The asymmetric unit contains two independent NOPCH_3_ molecules, which form a dimer linked by an N–H···O hydrogen bond (Figure 1). Although two molecules are present in the asymmetric unit, the corresponding geometrical parameters do not differ significantly (Table 1).

The conformation of both independent NOPCH_3_ molecules is not strictly planar. The NO_2_ group is slightly twisted with respect to the plane of the pyridine ring by 10.4(2)° in one molecule and 8.5(2)° in the second molecule, whereas the *N*-methylamino substituent at the 3-position is almost coplanar with the pyridine ring (Table 1).

The DFT-optimized geometry of NOPCH_3_ is consistent with the experimental structure in terms of the key bond lengths and angles. Selected DFT geometrical parameters are provided in Table 2, while complete details are collected in Table S1 (Supplementary Materials). Compared with the crystal structure, the main conformational difference concerns the nitro group, which is twisted in the solid state. This deviation can be attributed to intermolecular interactions present in the crystal lattice.

**Figure 2:**
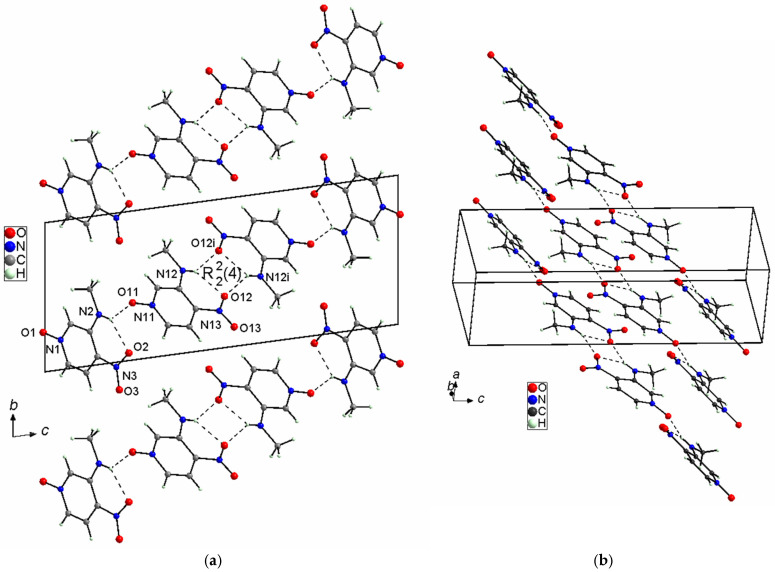
Packing diagram of NOPCH_3_ showing the tetramers with a graph of $R_{2}^{2}$ (**a**) and view of the stacking arrangement of the tetramers in the unit cell (**b**).

The 3-*N*-methylamino-4-nitropyridine-*N*-oxide molecules in the crystal interact with each other by electrostatic interactions between the opposite signs of the EP of molecules (Figure 1c). The three-dimensional molecular map of the electrostatic potential (3D-MESP) is related to the electron density within the molecule and is helpful in the nucleation and crystal growth processes. The calculated 3D MESP is mapped onto the total electron density isosurface (0.008 eÅ^−3^) and illustrates the size, shape, charge density, and the sites of the chemical reactivity. The intermolecular interactions between the oppositely signed EP lead to the formation of the intermolecular hydrogen bonds N–H···O, forming a dimeric structure with additional intramolecular N–H···O hydrogen bonds (Figure 1a). Such inversion-related dimers interact via bifurcated N–H···O hydrogen bonds forming a double dimeric structure or tetramer with a graph of $R_{2}^{2}$, which is almost parallel to the (104) crystallographic plane (Figure 2a).

In the crystal between the N–H···O hydrogen-bonded tetramers, there are no directional interactions; three are interacting mainly by the electrostatic interactions and van der Waals forces, forming a stacking structure (Figure 2b). However, within the stack, there are no π···π interactions between the pyridine rings since the distance between the gravity centers of the rings is too long for the effective interactions (Cgr···Cgr = 4.740(3) Å).

#### 2.1.2. Description of the Structure of 3-(*N*-3-Aminopropanoic-Acid)-4-Nitropyridine-*N*-Oxide (NOPCOOH)

**Figure 3:**
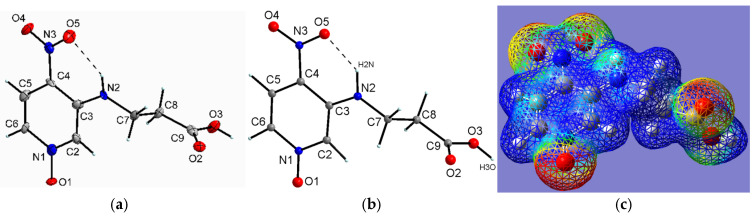
View of the X-ray structure of NOPCOOH (**a**), DFT-optimized molecule (**b**), and 3D MESP map of the molecule, color code of MESP maps is in the range of −0.05 (red) to 0.05 eÅ-1 (blue) (**c**). The displacement ellipsoids are shown at the 50% probability level, and H atoms are shown with arbitrary radii.

The 3-(*N*-3-aminopropanoic-acid)-4-nitropyridine-*N*-oxide (NOPCOOH) crystallizes in the non-centrosymmetric space group P212121 of the orthorhombic system with four molecules per unit cell. The asymmetric unit contains one 3-(*N*-3-aminopropanoic-acid)-4-nitropyridine-*N*-oxide molecule (Figure 3a). The nitro group is not coplanar with the pyridine ring but twisted. The dihedral angle between the planar pyridine ring and the NO_2_ group is equal to 9(2)°. The carbon skeleton substituent of the *N*-3-aminopropionic acid, together with the COOH group, is almost planar and forms an angle of 96.1(8)° with the plane of the pyridine ring. A quite similar conformation for the 3-(*N*-3-aminopropanoic-acid)-4-nitropyridine-*N*-oxide was obtained by DFT calculations (Figure 3b). Selected geometrical parameters (X-ray and DFT) are listed in Table 2, but full optimized parameters are listed in Table S2 (in the Supplementary Materials).

**Figure 4:**
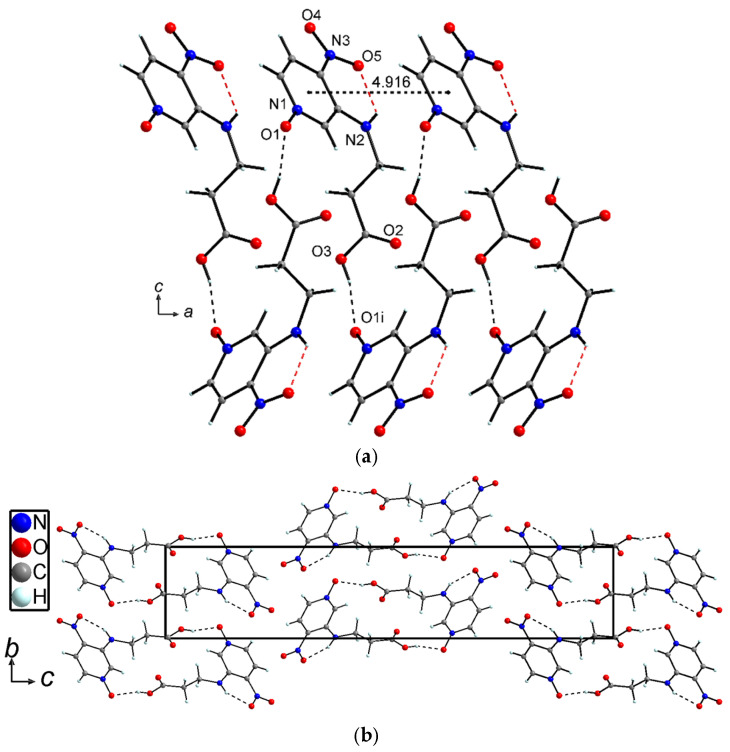
View of the O–H···O hydrogen-bonded helix (**a**) and the packing diagram in the unit cell (**b**) for NOPCOOH.

In the crystal, the molecules of 3-(*N*-aminopropionic acid)-4-nitropyridine-*N*-oxide related by a two-fold symmetry axis parallel to the a-axis are arranged into a helix connected by O–H···O hydrogen bonds running along the a-axis (Figure 4a). These hydrogen bonds form between the COOH group and the oxygen atom of the *N*-oxide of an adjacent molecule. The intermolecular interactions that lead to helix formation are well illustrated by the electrostatic potential of molecules (Figure 3c). The interaction between molecules becomes effective when they orient themselves with the parts of opposite potential signs relative to each other. Within the helix, there are no π···π interactions between the pyridine rings since the distance between the gravity centers of the rings is too long for the effective interactions (Cgr···Cgr = 4.9163 (6) Å).

These helices in the unit cell are related by the two-fold screw axes of the space group, forming the crystal structure. Between the helices, there are no directional interactions, such as hydrogen bonds; they are interacting mainly by the van der Waals forces and electrostatic interactions. These interactions are weaker than the hydrogen bonds that form helices; therefore, the (001) plane, as seen in Figure 4b, is the crystal cleavage plane.

The possible zwitterionic form of NOPCOOH was also considered. However, the X-ray geometry of the carboxylic group shows a clear differentiation between the C=O and C–OH bonds, with C9–O2 = 1.220(6) Å and C9–O3 = 1.340(6) Å. This bond-length pattern is consistent with a neutral carboxylic-acid group rather than a delocalized carboxylate anion. This interpretation is also supported by the presence of a carbonyl stretching band at 1709 cm^−1^ in the IR spectrum, so in the crystalline state, NOPCOOH is best described as a neutral carboxylic-acid species involved in strong intermolecular hydrogen bonding rather than as a fully zwitterionic carboxylate form.

### 2.2. Hirshfeld Surface Analysis

For a deeper analysis of intermolecular interactions in a crystal, analysis of the Hirshfeld surface (HS) [62] and two-dimensional fingerprint plots [63] have become an extremely useful method for gaining insight both qualitatively and quantitatively. For the analysis of hydrogen-bonded systems, d_norm_ mapping is best suited. According to the generally accepted color-coding scheme, red spots on HS represent short contacts, primarily strong hydrogen bonds, but weak hydrogen bonds (such as C–H···O) can also be identified because the sum of the di and de distances is much lower than the sum of the van der Waals radii of the participating atoms.

**Figure 5:**
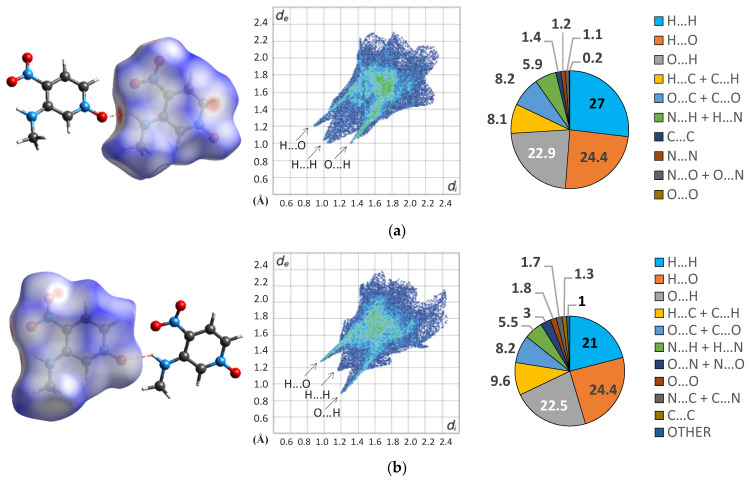
HS (**left**), the 2D fingerprint plots (**middle**), and the contributions of the respective intermolecular interactions (**right**) for both independent molecules in NOPCH_3_: (**a**) molecule containing N1, O1, and (**b**) molecule containing N11, O11 (for numbering see Figure 1).

Due to the presence of two crystallographically independent 3-*N*-methylamino-4-nitropyridine-*N*-oxide molecules in the NOPCH_3_ crystal, the HSs were generated separately for each (Figure 5). Mapping the dnorm on the Hirshfeld surface for both independent molecules of asymmetric unit 1 is used to compare the interactions between these molecules in the crystal. The red spots visible on the Hirshfeld surface, showing H····O/O···H contacts resulting from hydrogen-bonding interactions, the share of which on the HS is comparable for both independent molecules in the NOPCH_3_ crystal (67.3 and 66.9%). However, a quite significant difference in the HS is seen in the H···H interactions between both molecules of the asymmetric unit, which constitute 27 and 21%, respectively. Detailed deconvolution of 2D fingerprint plots for individual types of interactions for both independent molecules in the NOPCH_3_ crystal is given in Figure S1 (in Supplementary) and summarized in the circle diagram in Figure 5.

**Figure 6:**
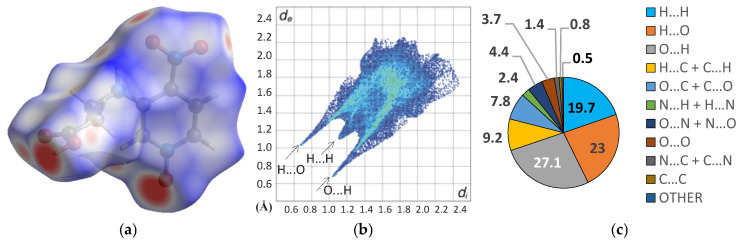
HS (**a**), the 2D fingerprint plots (**b**), and the contributions of the respective intermolecular interactions (**c**) for the NOPCOOH molecule.

The Hirshfeld surface illustrating the intermolecular contacts between 3-(*N*-aminopropionic acid)-4-nitropyridine-*N*-oxide molecules in NOPCOOH crystal was also mapped using the d_norm_ function. Owing to the relatively strong hydrogen bonds between the molecules in the NOPCOOH crystal, the red spots in the HS of the 3-(*N*-aminopropionic acid)-4-nitropyridine-*N*-oxide molecule are clearly visible (Figure 6).

As can be seen in Figure 6, larger areas on HS are visible around the OH group originating from the carboxyl group and the oxygen atom from the pyridine *N*-oxide group, and much smaller areas around both oxygen atoms of the nitro groups. These larger red spots correspond to H...O contacts resulting from O–H···O hydrogen bonding, resulting from interactions between 3-(*N*-aminopropionic acid)-4-nitropyridine-*N*-oxide molecules leading to the organization of the molecules into left-handed helices in the crystal along the a-axis. However, the much smaller red spots are formed because of intramolecular N–H···O contacts inside the helix around the oxygen atoms of the nitro group. The share of H...O/O...H contacts resulting from O–H···O hydrogen bonds in HS slightly exceeds 50% and is responsible for the stabilization of helices in the crystal. The interactions between the helices are mainly due to van der Waals forces, mainly H...H contact, which constitute nearly 20% of the HS of the molecule. The H…C/C…H and O..C/C…O contacts, which contribute to the HS 9.2% and 7.8%, respectively, also contribute to the interactions stabilizing the structure of the helices, as well as the interactions between them. The C...C contacts between aromatic pyridine rings are almost negligible (~0.4%) and confirm the absence of π···π interactions between them. Detailed deconvolution of 2D fingerprint plots for individual types of interactions between the 3-(*N*-aminopropionic acid)-4-nitropyridine-*N*-oxide molecules in NOPCOOH crystal is given in Figure S2 (in Supplementary Materials) and summarized in the circle diagram in Figure 6.

### 2.3. Vibrational Spectra

**Figure 7:**
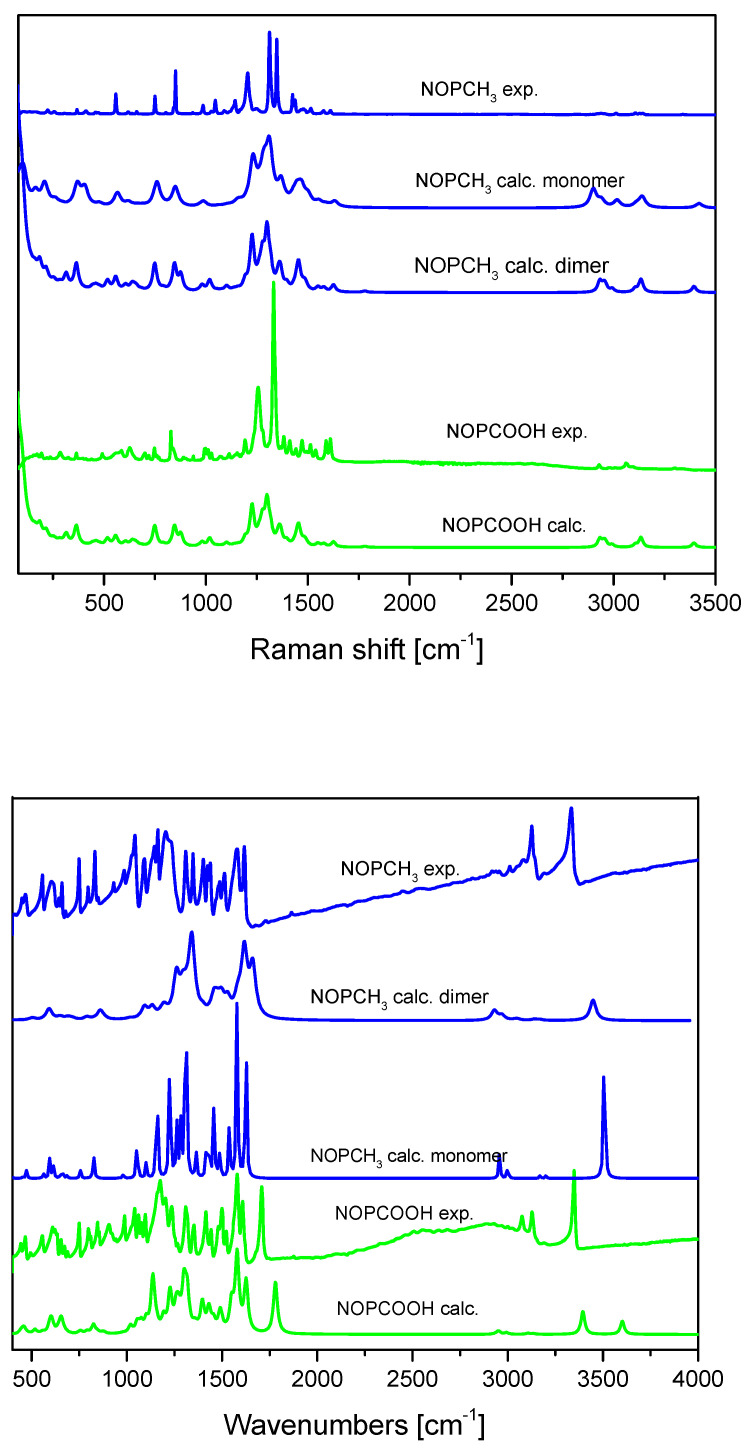
Comparison of the experimental and theoretical IR and Raman spectra of the studied derivatives.

The vibrational spectra of NOPCH_3_ and NOPCOOH were interpreted by considering the principal structural fragments present in the molecules: the pyridine *N*-oxide ring, the nitro group (NO_2_), the amino bridge (–NH–), and the substituent attached to the amino fragment, namely the methyl group in NOPCH_3_ and the propanoic-acid side chain in NOPCOOH. The assignments were based on comparison between the experimental FT-IR/Raman spectra, calculated normal modes, and literature data for related pyridine, nitropyridine, and pyridine *N*-oxide derivatives [24,25,26,27,28,29,30,33,35,36,37,40,64,65,66,67]. Because several calculated normal modes are coupled and involve simultaneous motion of more than one fragment, the assignments are given as dominant-mode descriptions rather than as purely isolated group vibrations. Selected diagnostic experimental and calculated bands are summarized in Table 3, whereas the complete vibrational assignment, including additional coupled modes and monomer/dimer calculated values for NOPCH_3_, is provided in Table S5. Infrared and Raman spectra of the studied NOPCH_3_ and NOPCOOH are shown in Figure 7.

Hydrogen bonding plays an important role in shaping several characteristic bands of NOPCOOH. The O–H vibrations of the carboxylic –COOH group are observed as a very broad and intense contour in the 2400–3000 cm^−1^ range, with weaker bands near 1278 cm^−1^ and 518 cm^−1^, corresponding to stretching, in-plane bending, and out-of-plane bending modes, respectively. The broad O–H stretching band is typical of strong hydrogen bonding and is commonly interpreted in terms of Fermi resonance between the stretching mode and an overtone of the bending vibration.

The –NH– bridge is present in both derivatives and can participate in intermolecular contacts involving oxygen atoms (in particular, nitro and/or *N*-oxide oxygen atoms) of neighboring molecules. The NH vibrations are observed in the 3349–3335 cm^−1^, 1500–1488 cm^−1^, and 670–658 cm^−1^ regions for stretching, in-plane bending, and out-of-plane bending modes, respectively.

Bands related to the alkyl/aliphatic fragments are also consistent with typical ranges reported for substituted pyridines. In NOPCH_3_, the methyl group gives rise to characteristic ν(CH_3_) bands, usually located between 2990 and 2950 cm^−1^ [64,65,66,67]. In the present spectra, the methyl stretching modes are assigned to $\nu_{as}$(CH_3_) at 3006 and 2998 cm^−1^ and $\nu_{s}$(CH_3_) at 2941 cm^−1^. The asymmetric bending $\delta_{as}$(CH_3_) contributes to bands in the 1500–1420 cm^−1^ range, while the symmetric bending $\delta_{s}$(CH_3_) occurs in the typical 1390–1370 cm^−1^ region. Additional methyl-related modes are observed in the following ranges: rocking 1045–1030 cm^−1^; ν(ϕ–CH3) 970–950 cm^−1^; γ(ϕ–CH_3_) 645–545 cm^−1^; and τ(CH_3_) 264–260 cm^−1^.

The characteristic bands associated with the N–O functionality of the pyridine *N*-oxide are observed in the 1240–1220 cm^−1^ range for stretching, 810–800 cm^−1^ for in-plane bending, and 320–290 cm^−1^ for out-of-plane bending modes.

Finally, the NO_2_ group contributes prominent bands (notably the symmetric and asymmetric stretching modes) whose positions may be sensitive to the local electronic environment and intermolecular interactions. Because NOPCOOH contains a strongly hydrogen-bonding carboxylic fragment and exhibits a different association pattern than NOPCH_3_, substituent-dependent changes in the NO_2_-related bands (Table 3) are expected and can be discussed as a manifestation of a modified NO_2_ environment in the solid state.

### 2.4. Electron Absorption and Emission Spectra

**Figure 8:**
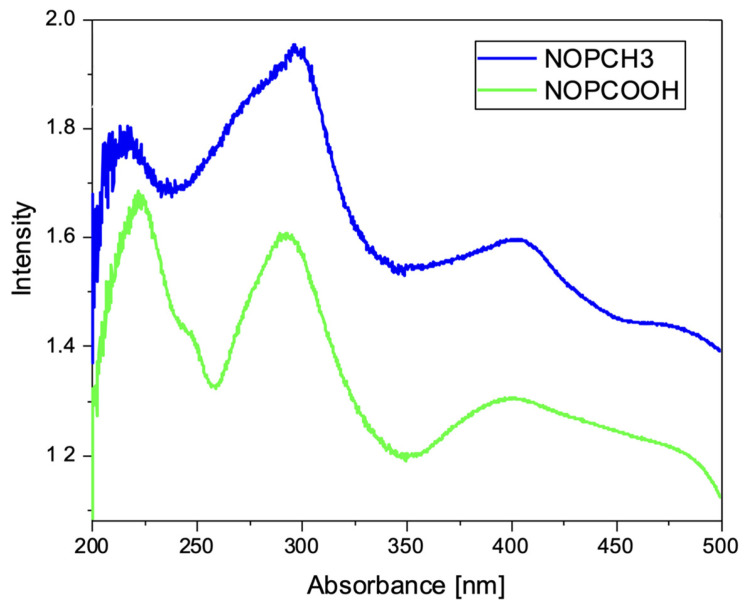
Diffuse-reflectance UV–Vis spectra of the studied samples are presented as qualitative pseudo-absorption profiles after baseline correction.

Band assignments were made based on earlier studies of related pyridine derivatives [32,39,40,41,68,69,70]. The diffuse-reflectance UV–Vis spectra converted using the Kubelka–Munk function are presented in Figure 8. The spectra are dominated by a broad absorption envelope in the 200–500 nm range. The calculated singlet and triplet excited-state energies, together with oscillator strengths for the singlet transitions and dominant one-electron orbital contributions, are summarized in Table 4. These calculations were used as qualitative support for the interpretation of the experimental spectra rather than as a fully quantitative simulation of the solid-state absorption profiles.

Calculated singlet states fall within the spectral range in which experimental absorption bands and shoulders are observed. In particular, the lowest singlet state S_1_ is predicted near 371 nm for both derivatives and exhibits a non-negligible oscillator strength. Therefore, S_1_ is included in the discussion of the long-wavelength absorption region rather than omitted from the spectral interpretation. Higher-energy singlet transitions with measurable oscillator strengths are also predicted in the near-UV region and can be associated qualitatively with the experimentally observed absorption features below approximately 320 nm.

**Figure 9:**
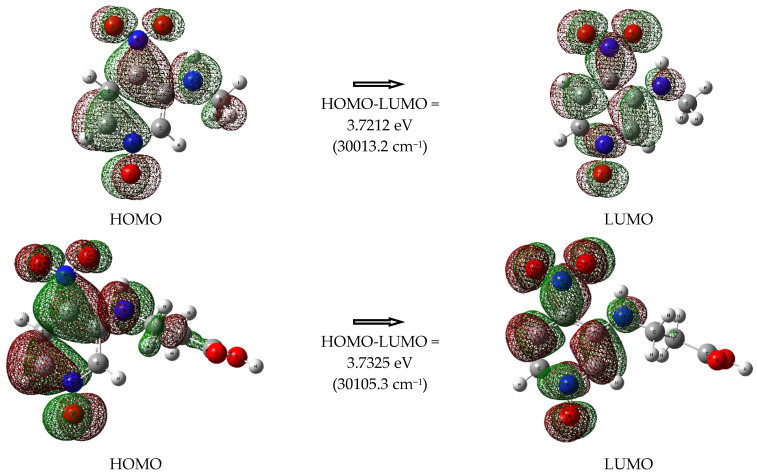
Frontier molecular orbitals HOMO and LUMO of NOPCH_3_ and NOPCOOH. The orbitals are shown as a qualitative visualization of the frontier electronic structure and should not be interpreted as representing all calculated electronic transitions.

Dominant one-electron orbital contributions obtained from the TD-DFT output are included in Table 4. This analysis shows that the calculated excited states should not be interpreted solely based on the HOMO–LUMO energy gap. Several singlet states contain mixed contributions from lower occupied and higher virtual orbitals, such as HOMO−1, HOMO−2, LUMO+1, or LUMO+2. Therefore, Figure 9 should be regarded only as a qualitative visualization of the frontier molecular orbitals, whereas the UV–Vis interpretation is based on the calculated excitation energies, oscillator strengths, and dominant orbital contributions summarized in Table 4. The calculated triplet-state energies are considered below only as energetic references for possible excited-state relaxation pathways, not as directly observed absorption transitions.

The orbital decomposition presented in Table 4 provides a more detailed basis for interpreting the experimental UV–Vis spectra than the HOMO–LUMO energy gap alone. In both derivatives, the lowest singlet transition S_1_ appears at approximately 371 nm and has a non-negligible oscillator strength. Its dominant contributions, HOMO→LUMO and HOMO−1→LUMO, indicate that the long-wavelength absorption edge involves the frontier π-electronic system of the nitropyridine *N*-oxide core with mixed contributions from two occupied orbitals. The most intense low-energy transition, S_4_, predicted near 316 nm for both NOPCH_3_ and NOPCOOH, also contains mixed HOMO/HOMO−1→LUMO character and therefore cannot be reduced to a single HOMO–LUMO excitation. This explains why the experimental near-UV absorption envelope should be discussed as a combination of several electronically related singlet transitions rather than as an isolated frontier-orbital transition.

The comparison of NOPCH_3_ and NOPCOOH shows that the calculated low-energy singlet states are very similar in energy and orbital character, indicating that the nitropyridine *N*-oxide chromophore is the dominant electronic unit responsible for the main absorption features. The amino substituent modifies the supramolecular organization and local hydrogen-bonding environment, but it does not fundamentally change the character of the lowest calculated singlet excitations. Differences between the experimental spectra of the two compounds should therefore be associated mainly with solid-state effects, hydrogen bonding, and local substituent-induced perturbations of the NO_2_/*N*-oxide environment rather than with a change in the primary chromophoric fragment. At higher energies, especially for S_6_–S_10_, the calculated transitions involve LUMO+1/LUMO+2 and deeper occupied orbitals, showing that the short-wavelength absorption region has a more complex orbital origin involving the pyridine ring, the nitro group, and the *N*-oxide functionality.

**Figure 10:**
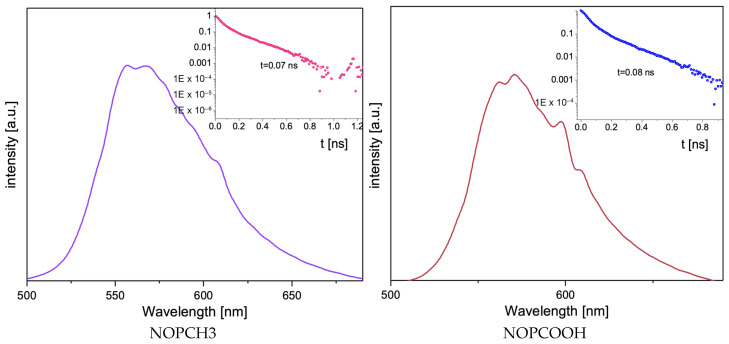
Emission spectra and decay curve of luminescence measured under excitation at 350 nm for the studied samples—NOPCH_3_ (**left**), NOPCOOH (**right**).

The emission spectra of the studied derivatives, together with the decay curves of the luminescence, are shown in Figure 10.

Spectra of the studied compounds contain a very broad band in the 500–700 nm range. The contour exhibits a maximum near ~560 nm (17,860 cm^−1^) and weak shoulders at approximately ~600 nm (16,670 cm^−1^) and ~610 nm (16,400 cm^−1^). The emission is markedly red-shifted relative to the main absorption region (26,500–45,000 cm^−1^), indicating substantial relaxation of the excited state prior to photon emission.

The calculated triplet-state energies listed in Table 4 fall partly within the energetic range of the observed visible emission. This energetic overlap suggests that triplet states may be accessible in the investigated molecules; however, it does not by itself provide sufficient evidence to assign the observed emission predominantly to phosphorescence or to a triplet-state origin. Such an assignment would require additional experimental verification, for example, oxygen-quenching experiments, temperature-dependent emission measurements, quantum-yield data, or optimized excited-state calculations including the T_1_ geometry and the adiabatic S_0_–T_1_ energy gap.

**Figure 11:**
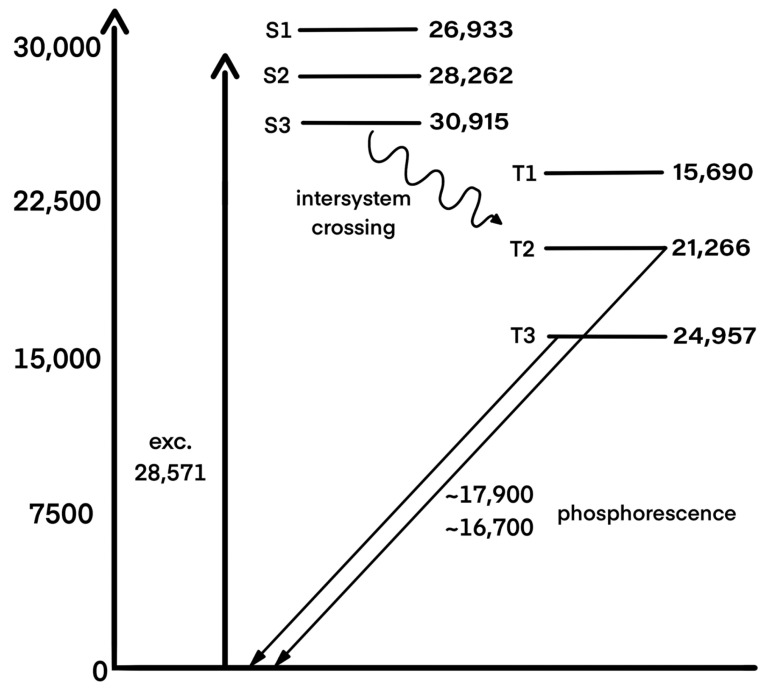
Qualitative Jablonski-type diagram of possible excited-state relaxation pathways in the studied derivatives.

Therefore, in the present work, the broad visible emission is interpreted conservatively as emission from a strongly relaxed excited-state manifold. A possible contribution of triplet states populated through intersystem crossing cannot be excluded, especially in view of the nitroaromatic character of the compounds, but the available experimental data do not allow a definitive separation of singlet- and triplet-derived contributions. On this basis, a qualitative Jablonski-type diagram is proposed in Figure 11 to summarize the possible excited-state relaxation pathways considered for the studied derivatives, following the general framework commonly used to describe molecular absorption, fluorescence, phosphorescence, internal conversion, and intersystem crossing [71,72,73,74,75]. The diagram includes absorption to singlet excited states, vibrational relaxation/internal conversion, possible intersystem crossing, fluorescence-like emission from relaxed singlet states, and a possible triplet-state contribution. The scheme is intended only as a qualitative representation of plausible relaxation pathways and does not imply a confirmed phosphorescence mechanism.

### 2.5. Solid-State ^1^H and ^13^C NMR Spectra

**Figure 12:**
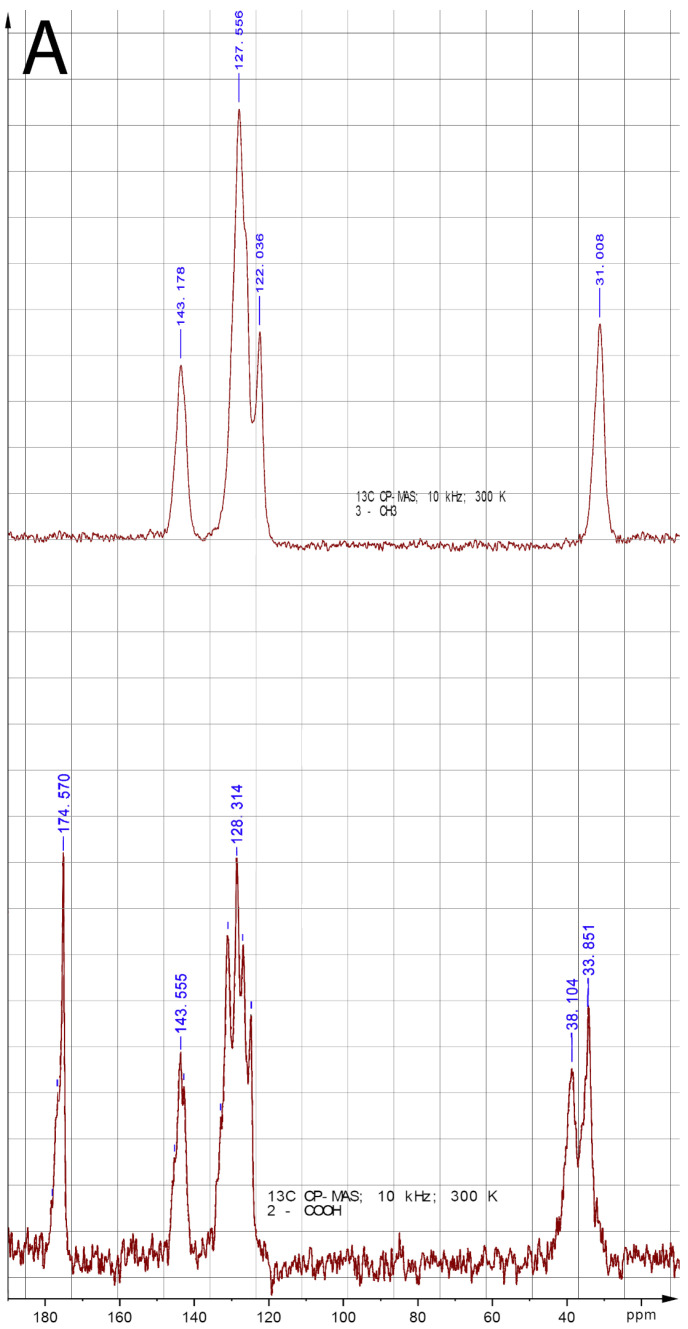

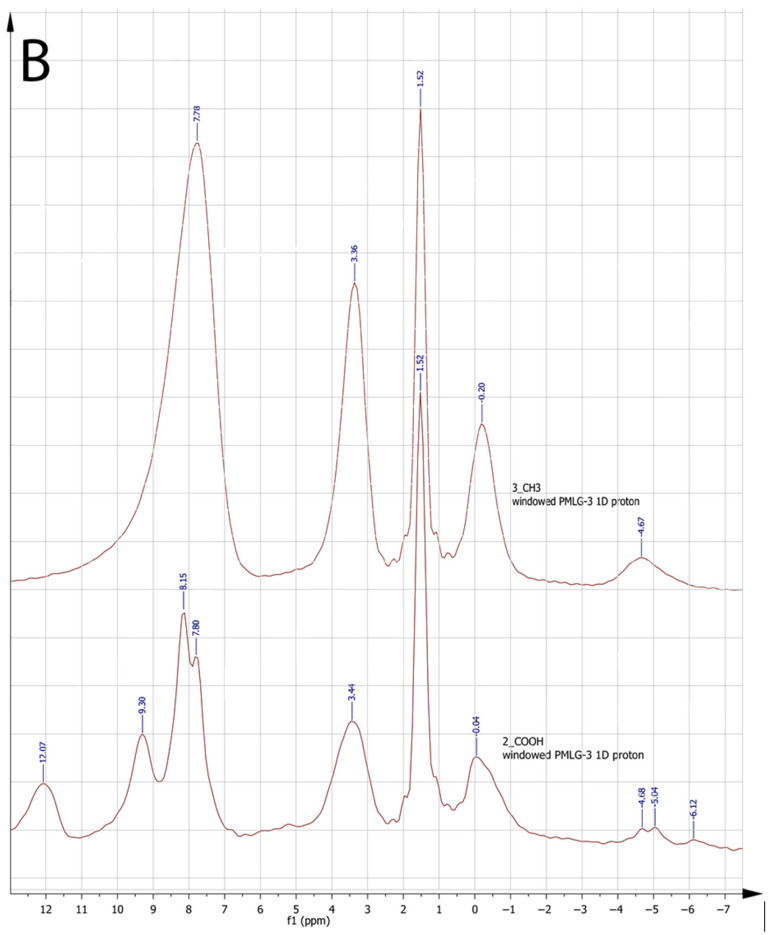
Solid-state ^13^C CP-MAS (**A**) and ^1^H WPMLG (**B**) NMR spectra of the studied NOPCH_3_ and NOPCOOH derivatives.

Solid-state ^1^H and ^13^C NMR spectra of the studied compounds are shown in Figure 12. The ^1^H spectra were recorded using the WPMLG (Windowed Phase-Modulated Lee-Goldburg) technique, whereas the ^13^C spectra were measured using the CP-MAS approach. Because the ^1^H spectra were acquired in the solid state with homonuclear decoupling, they were not treated as directly equivalent to conventional solution-state ^1^H NMR spectra. The assignment of the solid-state ^13^C CP-MAS and ^1^H WPMLG NMR spectra was guided by general NMR chemical-shift principles [76] and by literature data for pyridine, substituted pyridine, nitropyridine, and pyridine *N*-oxide derivatives [77,78,79,80,81,82,83,84,85,86]. These references provide comparative ranges for aromatic, methyl, methylene, *N*-oxide-related, and nitropyridine environments. In the present work, weak highly shielded features observed near 0 ppm or in the negative-ppm region of the processed ^1^H WPMLG spectra were not used for structural assignments.

The ^13^C CP-MAS NMR spectrum of NOPCH_3_ shows resonances in the aromatic region at 143.178, 127.556, 125.545, and 122.036 ppm, which are consistent with carbon atoms of the substituted pyridine *N*-oxide framework. An additional resonance at 31.008 ppm is assigned to the methyl carbon of the *N*-methylamino substituent. The number and positions of the observed ^13^C resonances support the proposed molecular constitution of NOPCH_3_, in agreement with the single-crystal X-ray structure.

The ^13^C CP-MAS spectra provide the most direct support for the carbon frameworks of both compounds, while the ^1^H WPMLG spectra are consistent with the presence of aromatic, hydrogen-bonded, and aliphatic proton environments. Resonances at 144.796, 143.555, 142.315, 132.390, 130.440, 128.314, 126.541, and 124.237 ppm are observed in the aromatic region and are assigned collectively to the pyridine *N*-oxide framework. The signals at 38.104 and 33.851 ppm are attributed to the two methylene carbons of the propanoic-acid side chain. The shoulders and partial splitting of selected ^13^C resonances most likely reflect the presence of slightly different local environments generated by hydrogen bonding and crystal packing in the solid state.

The solid-state ^1^H WPMLG NMR spectrum of NOPCH_3_ shows a broad resonance in the aromatic region centered at approximately 7.78 ppm, consistent with pyridine-ring protons. A further resonance near 3.36 ppm is attributed to the N–H proton of the methylamino fragment, while a signal in the aliphatic region near 1.52 ppm is compatible with the methyl group. A weak low-chemical-shift feature near 0.20 ppm and a minor feature in the negative-ppm region were also visible in the processed spectrum; however, these signals were not assigned to aromatic protons and were not used for structural interpretation. They are treated as non-diagnostic features of the solid-state ^1^H WPMLG spectrum, possibly related to baseline distortions, residual artifacts, scaling/referencing effects, or weak non-structural contributions.

The solid-state ^1^H WPMLG NMR spectrum of NOPCOOH contains downfield resonances at approximately 12.07 and 9.30 ppm, consistent with strongly hydrogen-bonded protons associated with the carboxylic-acid and/or N–H environments. Signals in the 8.15–7.80 ppm region are assigned collectively to pyridine-ring protons. Resonances in the aliphatic region, particularly near 3.44 and 1.52 ppm, are compatible with the methylene groups of the propanoic-acid side chain. A weak feature near 0.04 ppm and several negative-ppm features were present in the processed spectrum, but these signals were not assigned to pyridine-ring protons and were not used for structural interpretation.

Overall, the solid-state ^13^C CP-MAS and ^1^H WPMLG NMR data are consistent with the proposed structures of NOPCH_3_ and NOPCOOH. The ^13^C CP-MAS spectra provide the most direct support for the carbon frameworks of both compounds, while the ^1^H WPMLG spectra are consistent with the presence of aromatic, hydrogen-bonded, and aliphatic proton environments. The structural conclusions are therefore based on the combined evidence from solid-state NMR, single-crystal X-ray diffraction, and vibrational spectroscopy.

For the need of additional chemical characterization, solution-state ^1^H and ^13^C NMR spectra were also recorded for both derivatives and are provided in the Supplementary Materials (Figures S5–S8). For NOPCH_3_, the ^1^H NMR spectrum recorded in CDCl_3_ shows signals in the aromatic region at δ 8.02, 7.90, and 7.46 ppm, together with an N-methyl resonance at δ 3.01 ppm (Figure S5). The corresponding ^13^C NMR spectrum contains resonances at δ 143.22, 128.07, 127.56, 126.08, and 122.45 ppm in the aromatic carbon region, and a methyl-carbon resonance at δ 30.06 ppm (Figure S6). For NOPCOOH, the ^1^H NMR spectrum recorded in DMF-d_7_ shows aromatic-region resonances in the δ 8.46–7.58 ppm range and aliphatic-region signals compatible with the propanoic-acid side chain (Figure S7). The ^13^C NMR spectrum recorded in DMF-d_7_ shows resonances in the aromatic carbon region at δ 143.50, 128.71, 127.17, and 123.39 ppm, together with aliphatic-region signals (Figure S8). Because partial overlap with residual DMF-d_7_ signals and a broad water signal were observed for NOPCOOH, these solution-state NMR spectra were used as qualitative support for the proposed molecular constitution rather than for quantitative purity assessment or full individual signal assignment.

Experimental solid-state and solution-state NMR data were further compared with GIAO-calculated ^1^H and ^13^C chemical-shift regions for both derivatives (Table S6 in Supplementary Materials). The graphical GIAO-calculated ^1^H and ^13^C NMR spectra for the NOPCH_3_ dimer model are shown in Figures S3 and S4, respectively. For NOPCH_3_, the GIAO calculation performed for the hydrogen-bonded dimer model places the pyridine-ring protons in the δ 7.28–8.31 ppm range and the *N*-methyl protons in the δ 2.67–3.95 ppm range, in qualitative agreement with the solution-state ^1^H NMR spectrum recorded in CDCl_3_ and with the main solid-state ^1^H WPMLG spectral regions. The calculated ^13^C shifts also reproduce the expected separation between the aromatic carbon region (δ 128.1–149.6 ppm) and the methyl carbon (δ ca. 29.2 ppm). For NOPCOOH, the GIAO-calculated shifts place the aromatic protons in the δ 7.32–8.32 ppm range, the N–H proton at δ ca. 8.94 ppm, the side-chain methylene protons in the δ 2.41–3.55 ppm range, and the carboxylic proton at δ ca. 5.90 ppm. The corresponding calculated ^13^C shifts identify the carboxyl carbon at δ ca. 176.3 ppm, aromatic carbons in the δ 127.6–148.4 ppm range, and methylene carbons at δ ca. 32.5 and 40.4 ppm. These results support the assignment of the main spectral regions and are consistent with the experimental ^13^C CP-MAS and solution-state NMR data. Deviations for hydrogen-bonded protons are expected because the GIAO calculations were performed for finite molecular/cluster models and do not fully reproduce the periodic crystal environment and the complete hydrogen-bonding network.

## 3. Materials and Methods

### 3.1. Synthesis and Crystallization

**Scheme 2:**
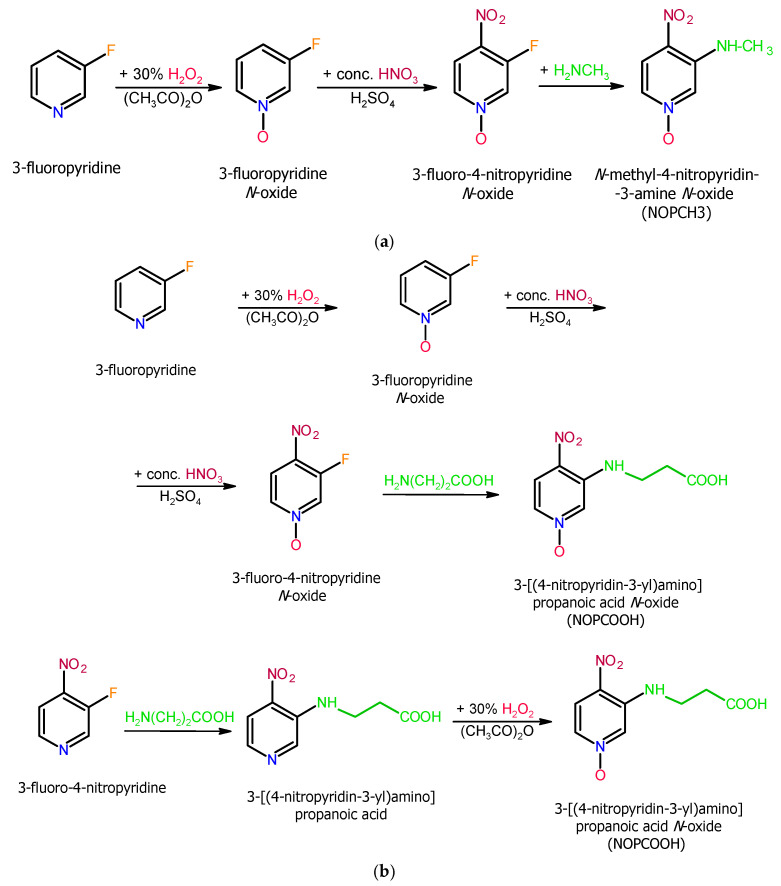
Synthetic routes to the studied nitropyridine amino *N*-oxide derivatives: (**a**) synthesis of 3-*N*-methylamino-4-nitropyridine *N*-oxide (NOPCH_3_) from 3-fluoro to 4-nitropyridine *N*-oxide by substitution with methylamine; (**b**) synthesis of [(4-nitropyridin-3-yl)amino]propanoic acid *N*-oxide (NOPCOOH) by substitution of 3-fluoro-4-nitropyridine with β-alanine, followed by oxidation to the corresponding pyridine *N*-oxide.

The synthetic routes leading to the studied derivatives are summarized in Scheme 2. NOPCH_3_ and NOPCOOH were obtained using two related, but not identical, synthetic routes. NOPCH_3_ was synthesized from the key intermediate 3-fluoro-4-nitropyridine *N*-oxide by nucleophilic substitution with methylamine. NOPCOOH was prepared by an alternative route involving nucleophilic substitution of 3-fluoro-4-nitropyridine with β-alanine, followed by oxidation of the pyridine nitrogen to the corresponding *N*-oxide. The final products were isolated as orange solids and used for further crystallographic and spectroscopic characterization.

Key intermediate 3-fluoro-4-nitropyridine *N*-oxide was prepared from 3-fluoropyridine according to a modified literature procedure [87,88,89,90]. 3-Fluoropyridine (3.00 g) was treated with 30% hydrogen peroxide (15 mL) and acetic anhydride (15 mL) at 50–60 °C. The resulting 3-fluoropyridine *N*-oxide was then subjected to nitration using a mixture of concentrated sulfuric acid (3 mL) and concentrated nitric acid (7 mL). After completion of the reaction, the reaction mixture was poured onto ice and neutralized with ammonium carbonate. The precipitated product was isolated and recrystallized from water to give 3-fluoro-4-nitropyridine *N*-oxide in 53% yield.

The 3-*N*-methylamino-4-nitropyridine *N*-oxide (NOPCH_3_) was obtained by nucleophilic substitution of the fluorine atom in 3-fluoro-4-nitropyridine *N*-oxide. 3-Fluoro-4-nitropyridine *N*-oxide (8.00 g) was suspended/dissolved in methanol (30 mL), and a 33% solution of methylamine in ethanol (20.0 g) was added. A vigorous exothermic reaction occurred, accompanied by immediate precipitation of an orange product. The reaction mixture was cooled, and the precipitated product was filtered off and recrystallized from a methanol/acetonitrile mixture. NOPCH_3_ was obtained as orange crystals; yield: 7.0 g (81.8%); m.p. 232 °C.

The [(4-Nitropyridin-3-yl)amino]propanoic acid *N*-oxide (NOPCOOH) was prepared by a two-step route. First, 3-fluoro-4-nitropyridine (0.71 g) was reacted with β-alanine (0.53 g) and sodium bicarbonate (0.84 g). β-Alanine and sodium bicarbonate were dissolved in water (5 mL), and a solution of 3-fluoro-4-nitropyridine in ethanol (15 mL) was added. The reaction mixture was heated under reflux for 15 min. Ethanol was then removed under reduced pressure, and the residue was slightly acidified with dilute hydrochloric acid. After cooling, the precipitated intermediate, 3-[(4-nitropyridin-3-yl)amino]propanoic acid, was filtered off, dried, and recrystallized from an ethanol/water mixture. The resulting substituted pyridine derivative was subsequently oxidized with 30% hydrogen peroxide in acetic anhydride to give the corresponding pyridine *N*-oxide, NOPCOOH. The final product was obtained as yellow crystals; yield: 70.5%; m.p. 208 °C with decomposition.

### 3.2. Single-Crystal X-Ray Data Collection for Nitropyridine Amino N-Oxide Derivatives: (NOPCH_3_) and (NOPCOOH)

Single-crystal X-ray diffraction intensity data for NOPCH_3_ and NOPCOOH were collected with graphite-monochromated Mo Kα radiation on a four-circle κ-geometry Xcalibur diffractometer equipped with a Sapphire2 CCD area detector (Rigaku Polska Sp. z o.o., Wroclaw, Poland). Data for NOPCH_3_ were collected at 295 K, whereas data for NOPCOOH were collected at 100 K (see Table 5).

Data collection, unit-cell refinement, data reduction and integration, as well as corrections for Lorentz and polarization effects and absorption, were performed using the CrysAlisPro 1.171.42.93a software system (Rigaku Polska Sp. z o.o., Wroclaw, Poland) [91]. Structure solution and refinement were carried out in Olex2 [92] using SHELXT-2014/7 for structure solution [93] and SHELXL-2018/3 for refinement [94]. Hydrogen atoms bonded to aromatic carbon atoms were placed in geometrically calculated positions and treated using a riding model with Uiso(H) = 1.2Ueq(C). The final difference Fourier maps showed no peaks of chemical significance. Details of data collection and refinement parameters are summarized in Table 5.

Molecular graphics were prepared using DIAMOND 3.0 [95]. Crystallographic data (CIF) for NOPCH_3_ and NOPCOOH are intended for deposition in the Cambridge Crystallographic Data Centre (CCDC); deposition numbers should be inserted in the final version in the standard CCDC statement format recommended by MDPI.

### 3.3. Hirshfeld Surface Analysis

Hirshfeld surface (HS) analysis assumes that electron density in a crystal is distributed over each molecule, so surfaces that encompass the spaces occupied by these molecules in the crystal can be defined. The HS can be defined by two distances: the internal distance, di, which defines the distance between the nearest atom inside the HS and the HS itself, and the external distance, de, which similarly describes the distance from the nearest atom outside the HS to the HS itself. Analyzing this data using appropriate functions (d_norm_, shape index, curvature) [96] provides important information about the interactions between molecules. To visualize the properties of the HS, these parameters are assigned a color scale, making specific interactions easily visible. The di values are plotted as a function of the de values, resulting in a two-dimensional (2D) fingerprint plot that graphically summarizes the interaction distances that affect the HS. Hirshfield surface analyses, and 2D fingerprint plots that were calculated using the Crystal Explorer Ver. 3.1 (University of Western Australia, Perth, Australia) program package [97]. Crystal structures were imported from CIF files. Hirshfeld surfaces were generated for each molecule using very high resolution and mapped with the d_norm_ function.

### 3.4. DFT Calculations

Molecular orbital calculations with full geometry optimization of NOPCH_3_ and NOPCOOH were performed with the Gaussian16 (Gaussian, Inc., Wallingford, CT, USA) program package [98]. All calculations were carried out using the DFT method (Becke3-Lee-Yang-Parr exchange-correlation functional B3LYP) [99,100,101] with the 6-31+G* basis set [102,103,104,105], assuming the geometry resulting from the X-ray diffraction study as the starting structures. As convergence criteria, the threshold limits of 0.00025 and 0.0012 a.u. were applied for the maximum force and the displacement, respectively.

TD-DFT calculations were performed for the optimized monomeric forms of NOPCH_3_ and NOPCOOH at the B3LYP/6-311G(2d,2p) level using the TD = (50-50, NStates = 10) keyword. This calculation provided ten singlet and ten triplet excited states for each derivative. Excitation energies, wavelengths, oscillator strengths for singlet transitions, and dominant one-electron orbital contributions were extracted from the Gaussian 16 output and used for the qualitative interpretation of the UV–Vis spectra.

In addition, GIAO NMR calculations were performed for the hydrogen-bonded NOPCH_3_ dimer model and for NOPCOOH using the B3LYP functional and the 6-311G(2d,2p) basis set. The calculations were carried out with Gaussian 16 using the NMR = GIAO keyword. Isotropic magnetic shielding was converted to calculated chemical shifts according to δcalc = σTMS − σsample, using the TMS reference shielding calculated at the same level of theory (σTMS(^1^H) = 31.8191 ppm and σTMS(^13^C) = 183.8006 ppm). The GIAO-calculated shifts were used as qualitative support for assigning the main ^1^H and ^13^C NMR spectral regions. Because the calculations were performed for molecular/cluster models rather than periodic crystal structures, they should not be regarded as full periodic simulations of the solid-state NMR spectra.

### 3.5. Spectroscopic Studies

IR spectra were recorded using a Nicolet iS50 FT-IR spectrometer (Thermo Fisher Scientific, Madison, WI, USA) equipped with an automated beamsplitter exchange system (iS50 ABX) and DLaTGS detectors for the mid-IR (KBr) and far-IR (solid substrate) regions. Polycrystalline mid-IR spectra were collected in the 4000–400 cm^−1^ range using Nujol mulls and KBr pellets. The spectra obtained using both sample preparations were identical, indicating that no chemical changes occurred in these matrices. The spectral resolution was set to 4 cm^−1^.

Raman spectra were measured in the 4000–80 cm^−1^ range in a backscattering geometry using an IFS Bruker 110/S spectrometer (Bruker, Ettlingen, Germany). The spectral resolution was set to 2 cm^−1^. A Nd:YAG laser operating at 1064 nm was used as the excitation source.

Electronic absorption spectra were measured in the 200–1500 nm range using a Cary Varian 5E UV–Vis–near-IR spectrophotometer (Varian, Inc., Palo Alto, CA, USA) equipped with a Praying Mantis diffuse-reflectance accessory. The baseline was recorded using Al_2_O_3_ powder as the reference material. The diffuse-reflectance spectra were converted to pseudo-absorption profiles using the Kubelka–Munk function, F(R) = (1 − R)^2^/2R, where R is the diffuse reflectance of the sample relative to the Al_2_O_3_ reference. The resulting F(R) spectra were used for qualitative comparison of the absorption features [106,107,108].

Excitation spectra were measured using an SSF-01 spectrofluorometer (SpectrLab Scientific Inc., Warsaw, Poland) equipped with PTI Felix software (Photon Technology International, Birmingham, NJ, USA) and an ASOC 10 arrangement. A 150 W xenon lamp (Hamamatsu Photonics K.K., Hamamatsu, Shizuoka, Japan) was used as the excitation source. The excitation spectra were corrected for variations in the excitation intensity.

Emission spectra were recorded using a femtosecond laser system (Coherent “Libra”) coupled to an optical parametric amplifier (Light Conversion “OperA”). The excitation beam was focused on the sample using a lens with a focal length of 15 cm, and the emission was collected in a direction perpendicular to the excitation beam. Appropriate long-pass filters were applied to eliminate unwanted radiation. Emission spectra and luminescence decay curves were recorded using a grating spectrograph (Princeton Instruments Acton 2500i, Princeton Instruments, Trenton, NJ, USA) coupled to a streak camera (Hamamatsu C5680, Hamamatsu Photonics K.K., Hamamatsu, Shizuoka, Japan), operating in the 200–1100 nm range with a temporal resolution of 20–100 ps. The luminescence properties of the studied compounds were investigated at room temperature.

Solid-state ^1^H NMR spectra were recorded on a JEOL ECZ500R 500 MHz spectrometer (JEOL Ltd., Tokyo, Japan) equipped with an AUTOMAS 3.2 mm probe. The samples were spun at the magic angle at 10 kHz, and spectra were acquired using the WPMLG (Windowed Phase-Modulated Lee-Goldburg) sequence to reduce homonuclear ^1^H–^1^H dipolar broadening and spinning-sideband contributions. Chemical shifts were reported relative to the TMS scale.

Solid-state ^13^C NMR spectra were recorded on a Bruker Avance III 300 MHz spectrometer (Bruker BioSpin GmbH, Rheinstetten, Germany) using a 4 mm CP-MAS probe. The samples were spun at the magic angle at 10 kHz and measured using the CP-MAS (Cross-Polarization Magic-Angle Spinning) technique.

Solution-state ^1^H and ^13^C NMR spectra were recorded at 300 K using deuterated solvents selected according to sample solubility. NOPCH_3_ was measured in CDCl_3_, whereas NOPCOOH was measured in DMF-d_7_. The ^1^H NMR spectra were recorded at 500.13 MHz, and the ^13^C NMR spectra were recorded at approximately 125.76–125.77 MHz. Chemical shifts were calibrated using residual solvent signals. The solution-state NMR spectra are provided in the Supplementary Materials as Figures S5–S8.

## 4. Conclusions

Two nitropyridine amino *N*-oxide derivatives, NOPCH_3_ and NOPCOOH, were synthesized via a multistep route from a fluoropyridine precursor involving oxidation to the corresponding pyridine *N*-oxide, nitration of the aromatic ring, and final nucleophilic substitution with methylamine or 3-aminopropanoic acid, respectively.

Single-crystal X-ray diffraction confirms the molecular structure of NOPCH_3_ and reveals a hydrogen-bonded dimer motif linked by an N–H···O interaction; the NO_2_ group is slightly twisted relative to the pyridine plane, consistent with packing-driven conformational effects.

The IR and Raman spectra, supported by DFT-based assignments, enable identification of characteristic modes of the pyridine *N*-oxide framework (N–O stretching/bending) and the key functional groups (NO_2_ and N–H in both derivatives; –COOH in NOPCOOH), using established vibrational–structure correlations.

For NOPCOOH, a very broad O–H stretching envelope in the 3000–2400 cm^−1^ region is consistent with strong hydrogen bonding typical of carboxylic acids; weaker bending modes further support the presence of a hydrogen-bonded –COOH motif in the solid state.

Diffuse reflectance UV–Vis spectra converted to pseudo-absorbance are dominated by bands in the 200–500 nm region. The TD-DFT calculations, including excitation energies, oscillator strengths, and dominant one-electron orbital contributions, provide qualitative support for the observed absorption features. The calculated transitions involve mixed contributions from frontier and deeper occupied/virtual orbitals; therefore, the spectra are discussed in terms of ligand-centered or mixed transitions involving the pyridine *N*-oxide, nitro group, and aromatic framework rather than being reduced to a simple HOMO–LUMO interpretation.

The photoluminescence spectra of both compounds exhibit a broad emission band in the visible range. The overlap between experimental emission energies and calculated triplet-state energies indicates that triplet states may be energetically accessible; however, the present data do not allow the emission to be assigned unambiguously to a triplet-state origin. Confirmation of any triplet contribution would require additional experiments, such as oxygen-quenching, temperature-dependent emission measurements, quantum-yield determination, or excited-state calculations including optimized T_1_ geometries.

The experimental solid-state and solution-state NMR data were additionally compared with GIAO-calculated ^1^H and ^13^C chemical-shift regions for both derivatives. The calculated regions support the assignment of the main aromatic, aliphatic, methyl, methylene, and carboxyl-carbon environments, while the graphical GIAO-calculated spectra for the NOPCH_3_ dimer model provide complementary support for the hydrogen-bonded dimer arrangement established by single-crystal X-ray diffraction. Because the calculations were performed for finite molecular/cluster models, they were used as qualitative support for the NMR interpretation rather than as full periodic simulations of the solid-state NMR spectra.

The presence of hard donor sites, including the *N*-oxide oxygen atom and, in NOPCOOH, an additional carboxylic-acid functionality, indicates that these compounds may serve as structurally characterized precursors for future coordination studies. In particular, the additional acid group in NOPCOOH may offer further coordination possibilities after deprotonation, but such coordination behavior was not investigated in the present work.

## Figures and Tables

**Table 1:** Selected geometrical parameters (Å, ^o^) for NOPCH_3_.

		X-Ray		DFT
		Molecule 1	Molecule 2	
N1—O1		1.297 (2)	1.284 (2)	1.264
C3—N2		1.337 (2)	1.337 (2)	1.347
C4—N3		1.424 (2)	1.427 (2)	1.436
N3—O2		1.227 (2)	1.242 (2)	1.245
N3—O3		1.230 (2)	1.232 (2)	1.227
O2—N3—O3		121.9 (2)	121.7 (2)	122.63
O2—N3—C3—C5		−10.1 (2)	−173.8 (2)	−179.95
C7—N2—C3—C4		169.0 (2)	8.2 (2)	179.99
O1—N1—C2—C3		−179.4 (2)	−177.3 (2)	179.99
Dihedral angle between the planes: NO_2_ (O2N3O3)/pyridine ring (N1, C2–C6)		10.4 (2)	8.5(2)	0.01
C3N2C7/pyridine ring (N1, C2–C6)		2.3 (2)	0.8(2)	0.01
Hydrogen-bond geometry (Å, ^o^) in crystal				
*D*—H···*A*	*D*—H	H···*A*	*D*···*A*	*D*—H···*A*
N2—H2···O2	0.839 (17)	2.038 (19)	2.665 (2)	131.0 (16)
N2—H2···O11	0.839 (17)	2.204 (17)	2.863 (2)	135.4 (17)
N12—H12···O12	0.816 (18)	2.046 (19)	2.654 (2)	131.0 (17)
N12—H12···O12 *^i^*	0.816 (18)	2.373 (18)	3.067 (2)	143.4 (18)
Symmetry code: (*i*) −*x*−1, −*y* + 1, −*z* + 1. Hydrogen-bond geometry (Å, ^o^) in gas-phase (DFT)				
N2—H···O2	1.009	1.862	2.642	131.56

**Table 2:** Selected geometrical parameters (Å,^o^) for NOPCOOH.

			X-Ray	DFT
N1—O1			1.313 (7)	1.263
C3—N2			1.360 (7)	1.350
C4—N3			1.415 (7)	1.438
C9—O2			1.220 (6)	1.204
C9—O3			1.340 (6)	1.351
N3—O4			1.248 (5)	1.226
N3—O5			1.249 (6)	1.245
O2—C9—O3			124.1 (5)	123.01
O4—N3—O5			121.3 (6)	122.59
C3—C4—N3—O4			169.9 (5)	−179.85
C2—C3—N2—C7			12.7 (8)	1.13
N2—C7—C8—C9			174.8 (6)	178.84
C7—C8—C9—O2			2.2 (9)	0.89
Dihedral angle between the planes: NO_2_ (O2N3O3)/pyridine ring (N1, C2–C6)			9.2 (8)	0.29
N2C7C8C9O2O3/pyridine ring (N1, C2–C6)			96.1 (8)	95.50
Hydrogen-bond geometry (Å, ^o^) in crystal				
*D*—H···*A*	*D*—H	H···*A*	*D*···*A*	*D*—H···*A*
N2—H2N···O1*^i^*	0.88	2.48	3.181 (6)	137
N2—H2N···O5	0.88	2.03	2.658 (6)	128
O3—H3o···N1*^ii^*	0.84	2.60	3.422 (5)	167
O3—H3o···O1*^ii^*	0.84	1.83	2.635 (5)	160
Symmetry codes: (*i*) x + 1, y + 1, z; (*ii*) x + 1/2, −y + 1/2, −z + 1. Hydrogen-bond geometry (Å, ^o^) in gas-phase (DFT)				
N2—H2N···O2	1.010	1.849	2.640	132.69

**Table 3:** Selected experimental and calculated vibrational bands of NOPCH_3_ and NOPCOOH.

Fragment/Mode	NOPCH_3_ calc./cm^−1^	NOPCH_3_ exp./cm^−1^	NOPCOOH calc./cm^−1^	NOPCOOH exp./cm^−1^
ν(N–H)	3448 (mon.); 3516/3507 (dim.)	3335	3599	3349
δ(N–H)	1618 (mon.); 1579/1574 (dim.)	1580	1579	1579
γ(N–H)	665 (mon.); 617 (dim.)	659	683	655
νas(NO_2_)	1525 (mon.); 1538/1521 (dim.)	1512	1549	1522
νs(NO_2_)	1341 (mon.); 1364 (dim.)	1348	1365	1353
δ(NO_2_)	860 (mon.); 844 (dim.)	832	846	847
ω(NO_2_)	762 (mon.); 661 (dim.)	749	756	750
ρ/γ(NO_2_)	497 (mon.); 472 (dim.)	468	462	467
ν(N–O)	1462 (mon.); 1457 (dim.)	1438	1455	1433
δ(N–O)	599 (mon.); 615 (dim.)	606	606	610
ν(C=O)	—	—	1778	1709
ν(O–H)_HB_	—	—	3602	broad 3000–2400
δ/γ(O–H···O)	—	—	1278; 1102; 846; 518	1236; 1097; 847; 495
ν(C–H) aromatic	3146; 3046	3127; 3085	3136	3107
ν(CH_3_)/ν(CH_2_)	2931; 2968	2920; 2957	2994	2951
ν(φ)	1659	1617	1626	1608
δ(C–H)	1452	1423	1432	1443
δ(CH_3_)/δ(CH_2_)	1459	1438	1482	1499

Note: Values are given in cm^−1^; ν = stretching; δ = in-plane bending; γ = out-of-plane bending; ω = wagging; ρ = rocking; φ = pyridine ring; ar = aromatic. For NOPCH_3_, calculated values are given as monomer/dimer values where applicable. NHO and NOH denote coupled vibrational modes involving N–H and oxygen-containing fragments and do not represent separate chemical functional groups; the full vibrational assignment is provided in Table S5.

**Table 4:** TD-DFT calculated singlet and triplet excited states of NOPCH_3_ and NOPCOOH (A–D), including excitation energies, wavelengths, oscillator strengths, and dominant orbital contributions.

**State**	**Energy/eV**	**λ/nm**	**ν/cm^−1^**	**f**	**Dominant One-Electron Orbital Contribution**
A. NOPCH_3_ singlet states					
S1	3.3371	371.54	26,916	0.0950	HOMO→LUMO (+0.505); HOMO−1→LUMO (+0.482)
S2	3.5012	354.12	28,239	0.0000	HOMO−2→LUMO (+0.685); HOMO−2→LUMO+2 (−0.144)
S3	3.8330	323.47	30,915	0.0000	HOMO−3→LUMO (+0.684); HOMO−3→LUMO+2 (+0.135)
S4	3.9243	315.94	31,652	0.2397	HOMO−1→LUMO (+0.490); HOMO→LUMO (−0.477); HOMO−1→LUMO+2 (+0.165)
S5	4.3945	282.14	35,444	0.0002	HOMO−5→LUMO (+0.687); HOMO−5→LUMO+2 (+0.132)
S6	4.8347	256.44	38,994	0.0312	HOMO→LUMO+1 (+0.594); HOMO−1→LUMO+2 (+0.340)
S7	5.2387	236.67	42,253	0.0229	HOMO→LUMO+2 (+0.514); HOMO−6→LUMO (−0.321); HOMO−4→LUMO (+0.251); HOMO−1→LUMO+1 (+0.231)
S8	5.4606	227.05	44,043	0.0420	HOMO→LUMO+2 (+0.442); HOMO−4→LUMO (−0.367); HOMO−1→LUMO+1 (−0.361); HOMO−6→LUMO (+0.135)
S9	5.5751	222.39	44,966	0.0000	HOMO−2→LUMO+2 (+0.674); HOMO−2→LUMO+1 (−0.162); HOMO−2→LUMO (+0.127)
S10	5.5903	221.79	45,089	0.0160	HOMO−1→LUMO+1 (+0.484); HOMO−4→LUMO (−0.355); HOMO−1→LUMO+2 (+0.309); HOMO−6→LUMO (+0.121)
B. NOPCOOH singlet states					
S1	3.3393	371.29	26,933	0.1021	HOMO→LUMO (+0.500); HOMO−1→LUMO (+0.488)
S2	3.5040	353.84	28,262	0.0001	HOMO−2→LUMO (+0.684); HOMO−2→LUMO+2 (−0.132)
S3	3.8330	323.46	30,915	0.0000	HOMO−3→LUMO (+0.681); HOMO−3→LUMO+2 (+0.121)
S4	3.9241	315.96	31,650	0.2353	HOMO−1→LUMO (+0.484); HOMO→LUMO (−0.482); HOMO−1→LUMO+2 (+0.166)
S5	4.3954	282.07	35,451	0.0002	HOMO−6→LUMO (+0.686)
S6	4.7878	258.96	38,616	0.0379	HOMO→LUMO+1 (+0.592); HOMO−1→LUMO+2 (+0.322); HOMO−4→LUMO (−0.131)
S7	4.9137	252.32	39,632	0.0011	HOMO−4→LUMO (+0.687); HOMO→LUMO+1 (+0.127)
S8	5.2397	236.62	42,261	0.0224	HOMO→LUMO+2 (+0.472); HOMO−1→LUMO+1 (+0.319); HOMO−7→LUMO (−0.297); HOMO−5→LUMO (+0.230)
S9	5.4182	228.83	43,701	0.0401	HOMO−1→LUMO+1 (+0.505); HOMO→LUMO+2 (−0.444); HOMO−5→LUMO (+0.180)
S10	5.5438	223.65	44,714	0.0362	HOMO−5→LUMO (+0.457); HOMO−1→LUMO+1 (−0.323); HOMO−1→LUMO+2 (−0.252); HOMO−7→LUMO (−0.196)
**State**	**Energy/eV**	**λ/nm**	**ν/cm^−1^**	**Dominant One-Electron Orbital Contribution**	
C. NOPCH_3_ selected triplet states					
T1	1.9403	638.98	15,650	HOMO→LUMO (+0.592); HOMO−1→LUMO (−0.353); HOMO→LUMO+2 (−0.133)	
T2	2.6299	471.43	21,212	HOMO−1→LUMO (+0.569); HOMO→LUMO (+0.377)	
T3	3.0926	400.91	24,944	HOMO−4→LUMO (+0.639); HOMO−4→LUMO+2 (+0.206); HOMO−6→LUMO (+0.149); HOMO−4→LUMO+1 (+0.145)	
D. NOPCOOH selected triplet states					
T1	1.9453	637.34	15,690	HOMO→LUMO (+0.589); HOMO−1→LUMO (−0.355); HOMO−1→LUMO+1 (+0.128); HOMO→LUMO+2 (−0.127)	
T2	2.6367	470.23	21,266	HOMO−1→LUMO (+0.566); HOMO→LUMO (+0.380)	
T3	3.0943	400.68	24,957	HOMO−5→LUMO (+0.638); HOMO−5→LUMO+2 (+0.187); HOMO−5→LUMO+1 (+0.169); HOMO−7→LUMO (+0.139)	

(A) Note for NOPCH_3_: HOMO = 44, LUMO = 45. Contributions are listed with Gaussian TD-DFT coefficients; only the largest components are shown for compactness. (B) Note for NOPCOOH: HOMO = 59, LUMO = 60. Contributions are listed with Gaussian TD-DFT coefficients; only the largest components are shown for compactness. (C,D) Triplet states are included as energetic references for possible excited-state relaxation pathways and are not treated as directly observed absorption transitions.

**Table 5:** Crystal data, data collection, and final refinement parameters for NOPCOOH and NOPCH_3_.

	NOPCH_3_	NOPCOOH
Formula	C_6_H_7_N_3_O_3_	C_8_H_9_N_3_O_5_
Molecular weight	169.15	227.18
Temperature (K)	295 (2)	100 (2)
Crystal system	triclinic	orthorhombic
Crystal color	orange	light-orange
Space group	$P\bar{1}$	*P2*_1_ *2*_1_ *2*_1_
*a* (Å)	4.7404 (2)	4.9163 (6)
*b* (Å)	8.0890 (4)	6.1461 (12)
*c* (Å)	19.1308 (9)	30.012 (4)
*α*, *β*, *γ* (^o^)	82.345 (4), 89.014 (4), 79.063 (4)	
*V* (Å^3^)	713.80 (6)	906.9 (2)
*Z*	4	4
*D_cal_* (g^.^cm^−3^)	1.574	1.664
θ range (^o^)	2.587–29.514	2.715–26.994
μ (mm^−1^)	0.129	0.141
Crystal size (mm)	0.344 × 0.142 × 0.106	0.382 × 0.052 × 0.042
T_min_/T_max_	0.9593/1.000	0.9771/1.000
Total/unique/obs. refls (*F*^2^ *> 2σ(F*^2^*)*)	12,345/3522/1968	3625/1874/1044
*R_int_*	0.0406	0.0723
*R* [*F*^2^ *> 2σ(F*^2^*)*] ^a^	0.0522	0.0755
*wR* [*F*^2^ all refls] ^a^	0.1085	0.0923
*S*	1.002	1.000
Flack parameter	-	−0.2 (10)
Δρ_max_, Δρ_min_ (eÅ^−3^)	+0.189, −0.182	+0.306, −0.353

^a^ R = Σ ||Fo|–|Fc||/ΣFo, wR = {Σ [w(Fo2–Fc2)2]/ΣwFo4}^½^; w–1 = σ2(Fo2) + (aP)2 where P = (Fo2 + 2Fc2)/3. The a parameter is 0.0390 for NOPCH_3_ and 0.0081 for NOPCOOH.

## Data Availability

The original contributions presented in this study are included in the article and Supplementary Materials. Further inquiries can be directed to the corresponding author.

## Supplementary Materials

The following supporting information can be downloaded at: https://www.mdpi.com/article/10.3390/ijms27146216/s1.
