# Supplementary material for: Structural and Spectroscopic Characterization of Two Nitropyridine Amino N-Oxide Derivatives
Source: Int J Mol Sci. 2026 Jul 12;27(14):6216. doi: 10.3390/ijms27146216 (PMC13410333; doi:10.3390/ijms27146216)
Supplement: Supplementary file 1 [file ijms-27-06216-s001.zip › ijms-4385394-supplementary.pdf]

Supplementary Materials

Structural and spectroscopic characterization of two nitropyridine amino N-oxide derivatives

Patrycja Godlewska[1], Wojciech Sąsiadek[1], Edyta Kucharska[1], Paulina Ropuszyńska-Robak[1], Lucyna Dymińska[1], Maciej Ptak[2], Jerzy Hanuza[2], Jan Janczak[2]

[1] *Department of Bioorganic Chemistry, Institute of Chemistry and Food Technology, Faculty of Engineering and Economy, University of Economics, Komandorska 118/120, 53 - 345 Wrocław, Poland,*

[2] *Institute of Low Temperature and Structure Research, Polish Academy of Sciences, Okólna 2, 50-950 Wrocław, Poland*

Contents

Table S1. DFT-optimized structural parameters of 3-N-methylamino-4-nitropyridine N-oxide ($NOPCH_3$)
Table S2. DFT-optimized structural parameters of [(4-nitropyridin-3-yl)amino]propanoic acid N-oxide (NOPCOOH)
Table S3. Solid-state NMR acquisition parameters for $NOPCH_3$ and NOPCOOH
Table S4. Experimental solid-state NMR spectral features of $NOPCH_3$ and NOPCOOH
Table S5. Full experimental and calculated vibrational assignments for $NOPCH_3$ and NOPCOOH
Table S6. Experimental and GIAO-calculated ^{1}H and ^{13}C NMR chemical-shift regions for $NOPCH_3$ and NOPCOOH
Figure S1. Deconvolution of the 2D fingerprint plots for the individual intermolecular contacts in both crystallographically independent molecules of the $NOPCH_3$ crystal
Figure S2. Deconvolution of the 2D fingerprint plots for the individual intermolecular contacts in the NOPCOOH crystal
Figure S3. Calculated ^{1}H NMR spectrum of the hydrogen-bonded $NOPCH_3$ dimer model
Figure S4. Calculated ^{13}C NMR spectrum of the hydrogen-bonded $NOPCH_3$ dimer model
Figure S5. Solution-state ^{1}H NMR spectrum of $NOPCH_3$ recorded in $CDCl_3$.
Figure S6. Solution-state ^{13}C NMR spectrum of $NOPCH_3$ recorded in $CDCl_3$.
Figure S7. Solution-state ^{1}H NMR spectrum of NOPCOOH recorded in DMF-d_7.
Figure S8. Solution-state ^{13}C NMR spectrum of NOPCOOH recorded in DMF-d_7.

Table S1. DFT-optimized structural parameters of 3-N-methylamino-4-nitropyridine N-oxide ($NOPCH_3$).

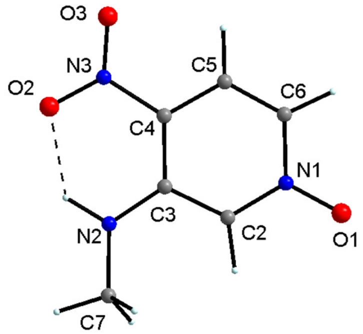

Bonds (Å)					
O1—N1	1.263	N1—C2	1.358	C2—C3	1.404
C3—C4	1.427	C4—C5	1.396	C5—C6	1.362
C6—N1	1.381	C3—N2	1.347	N2—C7	1.447
C4—N3	1.436	N3—O2	1.245	N3—O3	1.227
C2—H	1.075	C5—H	1.078	C6—H	1.075
N2—H	1.009	C7—H	1.093		
**Angles (°)**					
O1—N1—C2	120.61	O1—N1—C6	119.74	N1—C2—C3	123.05
C2—C3—C4	116.47	C3—C4—C5	119.23	C4—C5—C6	121.60
C5—C6—N1	120.00	C6—N1—C2	119.65	C2—C3—N2	120.05
C3—N2—C7	124.52	N2—C3—C4	123.48	C3—C4—N3	123.08
C5—C4—N3	117.69	C4—N3—O2	118.70	C4—N3—O3	118.67
O2—N3—O3	122.63				

Torsion angles (°)			
O1—N1—C2—C3	179.99	N1—C2—C3—C4	0.01
C2—C3—C4—C5	-0.01	C3—C4—C5—C6	0.00
C4—C5—C6—N1	-0.01	C5—C6—N1—C2	-0.02
N1—C2—C3—N2	-179.99	C2—C3—N2—C7	0.01
C7—N2—C3—C4	-179.99	N2—C3—C4—C5	179.99
N2—C3—C4—N3	-0.02	C3—C4—N3—O2	0.06
C3—C4—N3—O3	-179.94		

Hydrogen bonding geometry

D—H···A	*D—H*	*H···A*	*D···A*	*D—H··A*
N2—H···O2	1.009	1.862	2.642	131.56

Table S2. DFT-optimized structural parameters of [(4-nitropyridin-3-yl)amino]propanoic acid N-oxide (NOPCOOH).

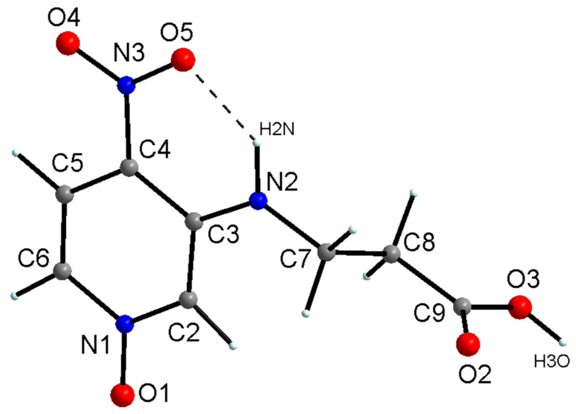

Bonds (Å)					
O1—N1	1.263	N1—C2	1.358	C2—C3	1.404
C3—C4	1.427	C4—C5	1.396	C5—C6	1.361
C6—N1	1.381	C3—N2	1.350	N2—C7	1.451
C7—C8	1.533	C8—C9	1.510	C9—O2	1.204
C9—O3	1.351	C4—N3	1.438	N3—O4	1.226
N3—O5	1.245	C2—H	1.075	C5—H	1.078
C6—H	1.075	C7—H	1.089	C8—H	1.091

Angles (°)					
O1—N1—C2	120.57	O1—N1—C6	119.75	N1—C2—C3	123.17
C2—C3—C4	116.28	C3—C4—C5	119.32	C4—C5—C6	121.64
C5—C6—N1	119.91	C6—N1—C2	119.68	C2—C3—N2	120.62
C3—N2—C7	125.89	N2—C7—C8	113.16	C7—C8—C9	111.77
C8—C9—O2	125.27	C8—C9—O3	118.70	C4—N3—O3	111.72
O2—C9—O3	123.01	C3—C4—N3	123.19	C5—C4—N3	117.49
C4—N3—O4	118.63	C4—N3—O5	118.78	O4—N3—O5	122.59

Torsion angles (°)			
O1—N1—C2—C3	-179.90	N1—C2—C3—C4	0.49
C2—C3—C4—C5	0.44	C3—C4—C5—C6	-0.17
C4—C5—C6—N1	-0.08	C5—C6—N1—C2	0.04
N1—C2—C3—N2	179.07	C2—C3—N2—C7	1.33
C3—N2—C7—C8	82.79	N2—C7—C8—C9	178.84
C7—C8—C9—O2	0.89	C7—C8—C9—O3	-179.09
C2—C3—C4—N3	-179.58	N2—C3—C4—N3	0.83
C3—C4—N3—O4	-179.86	C3—C4—N3—O5	0.25

Hydrogen bonding geometry				
D—H···A	*D—H*	*H···A*	*D···A*	*D—H··A*
N2—H2N···O5	1.010	1.849	2.640	132.69

Table S3. Solid-state NMR acquisition parameters for $NOPCH_3$ and NOPCOOH

Parameter	^1H WPMLG NMR	^{13}C CP-MAS NMR
Spectrometer	JEOL ECZ500R	Bruker Avance III
Operating frequency	500 MHz	300 MHz
Probe	AUTOMAS 3.2 mm	4 mm CP-MAS
MAS rate	10 kHz	10 kHz
Pulse sequence	WPMLG	CP-MAS

Table S4. Experimental solid-state NMR spectral features of $NOPCH_3$ and NOPCOOH.

Compound	Nucleus / technique	δ / ppm	Assignment / interpretation and comment
$NOPCH_3$	^{13}C CP-MAS	143.178, 127.556, 125.545, 122.036	Pyridine N-oxide carbon framework; assigned collectively to aromatic carbons.
$NOPCH_3$	^{13}C CP-MAS	31.008	Methyl carbon of the N-methylamino substituent.
$NOPCH_3$	^1H WPMLG	ca. 7.78	Aromatic proton region; main diagnostic aromatic-region feature.
$NOPCH_3$	^1H WPMLG	ca. 3.36	N–H region; assigned conservatively.
$NOPCH_3$	^1H WPMLG	ca. 1.52	Aliphatic region; compatible with methyl protons.
$NOPCH_3$	^1H WPMLG	ca. 0.20 and negative-ppm feature	Not assigned; not used for structural interpretation.
NOPCOOH	^{13}C CP-MAS	177.583, 176.165, 174.570	Carboxyl-carbon region; main resonance with weak shoulders.
NOPCOOH	^{13}C CP-MAS	144.796, 143.555, 142.315, 132.390, 130.440, 128.314, 126.541, 124.237	Pyridine N-oxide carbon framework; assigned collectively to aromatic carbons.
NOPCOOH	^{13}C CP-MAS	38.104, 33.851	Methylene carbons of the propanoic-acid side chain.
NOPCOOH	^1H WPMLG	ca. 12.07, 9.30	Strongly hydrogen-bonded proton environments; COOH/N–H region.
NOPCOOH	^1H WPMLG	ca. 8.15–7.80	Aromatic proton region; pyridine-ring protons.
NOPCOOH	^1H WPMLG	ca. 3.44, 1.52	Aliphatic proton region; propanoic-acid side chain.
NOPCOOH	^1H WPMLG	ca. 0.04 and negative-ppm features	Not assigned; not used for structural interpretation.

Table S5. Full experimental and calculated vibrational assignments for NOPCH$_3$ and NOPCOOH.

Fragment	Mode	NOPCH$_3$ calc. monomer / cm^{-1}	NOPCH$_3$ calc. dimer / cm^{-1}	NOPCH$_3$ exp. / cm^{-1}	NOPCOOH calc. / cm^{-1}	NOPCOOH exp. / cm^{-1}	Comment
NH	ν(N–H)	3448 νNH, νNH···O	3516 νs(NH); 3507 νas(NH)	3335	3599 νNH	3349	N–H stretching; dimer values for NOPCH$_3$ reflect two N–H environments.
NH	δ(N–H)	1618 δNH	1579 δs(NH); 1574 δas(NH···O)	1580	1579 δNH	1579	N–H bending; coupled with H-bonding in the dimer.
NH	γ(N–H)	665 γNH	617 γNH	659	683 γNH	655	N–H out-of-plane motion.
NO$_2$	νas(NO$_2$)	1525 νas(NO$_2$)	1538 νs(NO$_2$/H); 1521 νas(NO$_2$/H)	1512	1549 νas(NO$_2$)	1522	The original NOH label denotes coupled NO$_2$/N–H motion, not a separate NOH group.
NO$_2$	νs(NO$_2$)	1341 νs(NO$_2$)	1364 νs(NO$_2$) + δ(NH···O)	1348	1365 νs(NO$_2$)	1353	NO$_2$ symmetric stretching.
NO$_2$	δ(NO$_2$)	860 δNO$_2$	844 δNO$_2$	832	846 δNO$_2$	847	NO$_2$ deformation.
NO$_2$	ω(NO$_2$)	762 ωNO$_2$	661 ω(NO$_2$/H)	749	756 ωNO$_2$	750	NO$_2$ wagging; coupled in NOPCH$_3$ dimer.
NO$_2$	ϱ/γ(NO$_2$)	497 γNO$_2$	472 γ(NO$_2$/H)	468	462 ϱNO$_2$	467	NO$_2$ rocking/out-of-plane deformation.
N-oxide	ν(N–O)	1462 νNO	1457 ν(NO···H)	1438	1455 νNO	1433	Pyridine N-oxide N–O stretching.
N-oxide	δ(N–O)	599 δNO	615 δNO	606	606 δNO	610	N–O bending.
Combination	ν(φ–NO$_2$)	1131 ν(φ–NO$_2$)	1131 νφ + νNO + νCN	1093	1226 ν(φ–NO$_2$)	1235	Mode involving pyridine ring and nitro substituent.
Combination	ν(NO$_2$–φ–NO)	371 ν(NO$_2$–φ–NO)	370 ν(NO–φ–NO/H)	434	365 ν(NO$_2$–φ–NO)	420	Mixed low-frequency ring/substituent mode.
COOH	ν(O–H)	—	—	—	3602 νOH	broad 2400–3000; weak component near 3590	Strongly hydrogen-bonded carboxylic O–H stretching envelope.
COOH	ν(C=O)	—	—	—	1778 νC=O	1709	Carboxylic-acid carbonyl stretching.
COOH	δ(O–H···O)	—	—	—	1278 δOH···O; 1102 δOH···O	1236; 1097	In-plane bending of H-bonded COOH.
COOH	γ(O–H···O)	—	—	—	846 γOH···O; 518 γOH···O	847; 495	Out-of-plane H-bonded COOH modes.
COOH	δ(C=O)	—	—	—	1137 δC=O	1097	C=O deformation / coupled COOH mode.
Aromatic / aliphatic	ν(C–H)	3146; 3046	—	3127; 3085	3136	3107	Aromatic C–H stretching.
Aromatic / aliphatic	ν(CH$_2$)	—	—	—	2994	2951	Methylene stretching in propanoic-acid side chain.
Aromatic / aliphatic	ν(CH$_3$)	2931; 2968	—	2920; 2957	—	—	Methyl stretching in NOPCH$_3$.
Aromatic / aliphatic	ν(φ)	1659	—	1617	1626	1608	Pyridine-ring stretching.
Aromatic / aliphatic	δ(C–H)	1452	—	1423	1432	1443	Aromatic C–H bending.
Aromatic / aliphatic	δ(CH$_2$)	—	—	—	1482	1499	Methylene bending.
Aromatic / aliphatic	δ(CH$_3$)	1459	—	1438	—	—	Methyl bending.

Note: Abbreviated labels retained from the calculated normal-mode assignments are explained in the legend above. The labels NHO and NOH denote coupled motions involving N–H/O–H and N–O/NO$_2$ coordinates in hydrogen-bonded environments; they do not denote independent chemical groups. All wavenumbers are given in cm^{-1}.

Table S6. Experimental and GIAO-calculated ^{1}H and ^{1}[illegible]C NMR chemical-shift regions for $NOPCH_3$ and NOPCOOH

Compound	Nucleus / environment	Solution-state NMR δ / ppm	Solid-state NMR δ / ppm	GIAO-calculated δ / ppm	Assignment / comment
$NOPCH_3$	^{1}H; pyridine-ring H	8.02, 7.90, 7.46 ($CDCl_3$)	ca. 7.78	7.28–8.31	Aromatic/pyridine proton region.
$NOPCH_3$	^{1}H; N–CH_3	3.01 ($CDCl_3$)	ca. 1.52	2.67–3.95	Methyl protons; solid-state value reflects the broad aliphatic-region feature.
$NOPCH_3$	^{1}H; N–H / H-bonded proton	not assigned	ca. 3.36, assigned conservatively	ca. 9.33	Highly sensitive to hydrogen bonding and model geometry; used only as qualitative support.
$NOPCH_3$	^{13}C; pyridine-ring C	143.22, 128.07, 127.56, 126.08, 122.45 ($CDCl_3$)	143.178, 127.556, 125.545, 122.036	128.1–149.6	Aromatic/pyridine N-oxide carbon framework.
$NOPCH_3$	^{13}C; N–CH_3	30.06 ($CDCl_3$)	31.008	ca. 29.2	Methyl carbon of the N-methylamino substituent.
NOPCOOH	^{1}H; pyridine-ring H / N–H region	8.46–7.58 (DMF-d_7), partially overlapped	12.07, 9.30, and 8.15–7.80	7.32–8.32; N–H ca. 8.94	Aromatic and hydrogen-bonded proton environments; solution signals partly overlap with residual DMF-d_7.
NOPCOOH	^{1}H; CH_2 side chain	aliphatic-region signals; partly overlapped with DMF-d_7/water	ca. 3.44 and 1.52	2.41–3.55	Methylene protons of the propanoic-acid side chain; interpreted qualitatively.
NOPCOOH	^{1}H; COOH proton	not reliably assigned	12.07 / 9.30 region, collectively hydrogen-bonded	ca. 5.90	Strongly affected by hydrogen bonding; calculated molecular value is not expected to reproduce the full solid-state H-bonding environment.
NOPCOOH	^{13}C; C=O	not reliably assigned because of DMF-d_7 overlap	177.583, 176.165, 174.570	ca. 176.3	Carboxyl carbon region.
NOPCOOH	^{13}C; pyridine-ring C	143.50, 128.71, 127.17, 123.39 (DMF-d_7)	144.796, 143.555, 142.315, 132.390, 130.440, 128.314, 126.541, 124.237	127.6–148.4	Aromatic/pyridine N-oxide carbon framework.
NOPCOOH	^{13}C; CH_2 side chain	aliphatic-region signals; partly overlapped with DMF-d_7	38.104, 33.851	40.4, 32.5	Methylene carbons of the propanoic-acid side chain.

Note: The GIAO-calculated chemical shifts were obtained from isotropic magnetic shieldings using $\delta calc = \sigma TMS - \sigma sample$, with $\sigma TMS(^{1}H) = 31.8191$ ppm and $\sigma TMS(^{13}C) = 183.8006$ ppm. The values are used as qualitative support for assignment of the main spectral regions. Because finite molecular/cluster models were used rather than periodic crystal structures, the calculated values should not be interpreted as a complete simulation of the solid-state CP-MAS/WPMLG spectra. For NOPCOOH, solution-state NMR signals partially overlap with residual DMF-d_7 and water signals; therefore, they are used qualitatively and not for quantitative purity assessment.

Figure S1. Deconvolution of the 2DFP plots for the individual interactions for both crystallographically independent molecules in the $NOPCH_3$ crystal.

(a) Molecule containing N2 and O1

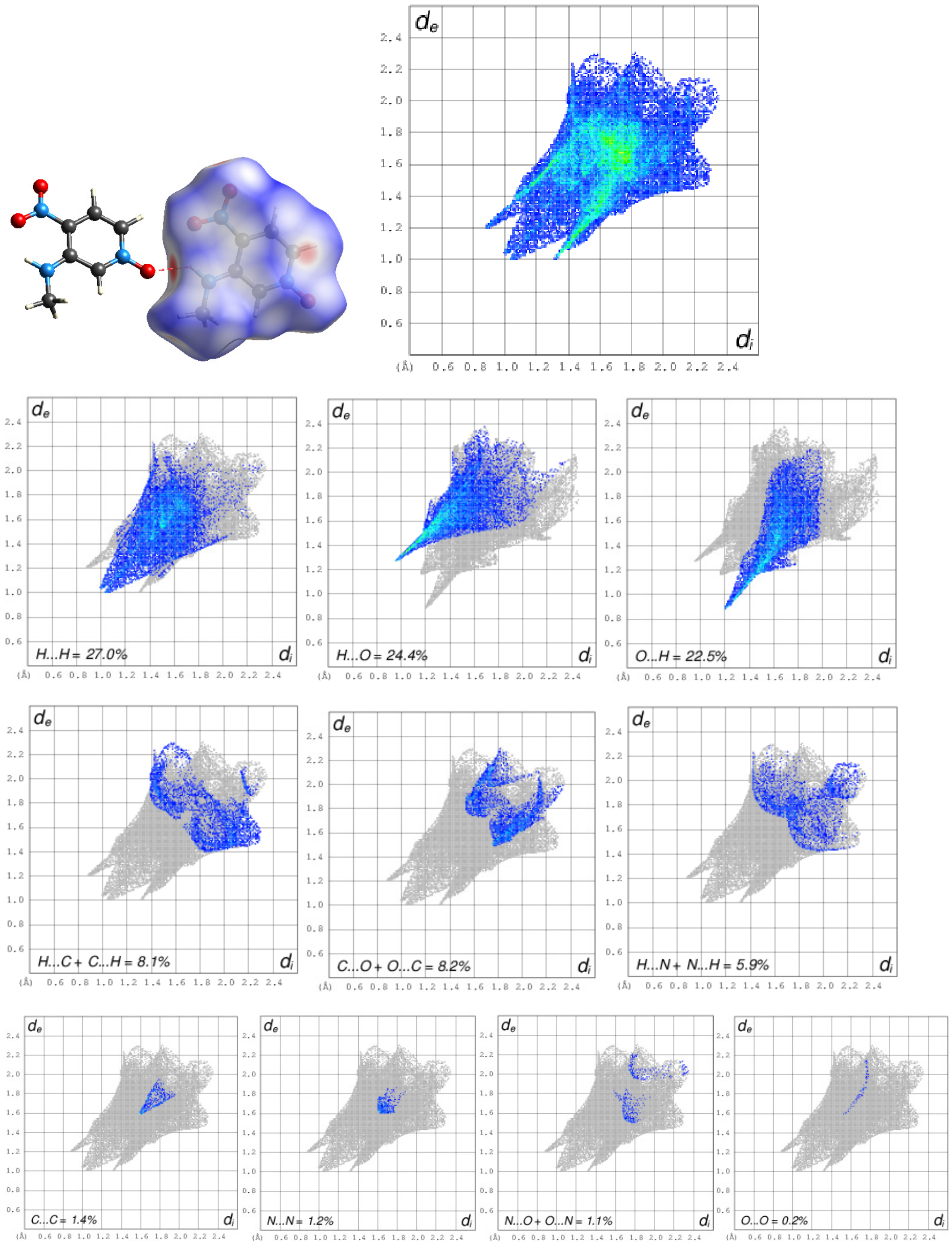

(b) Molecule containing N11 and O11

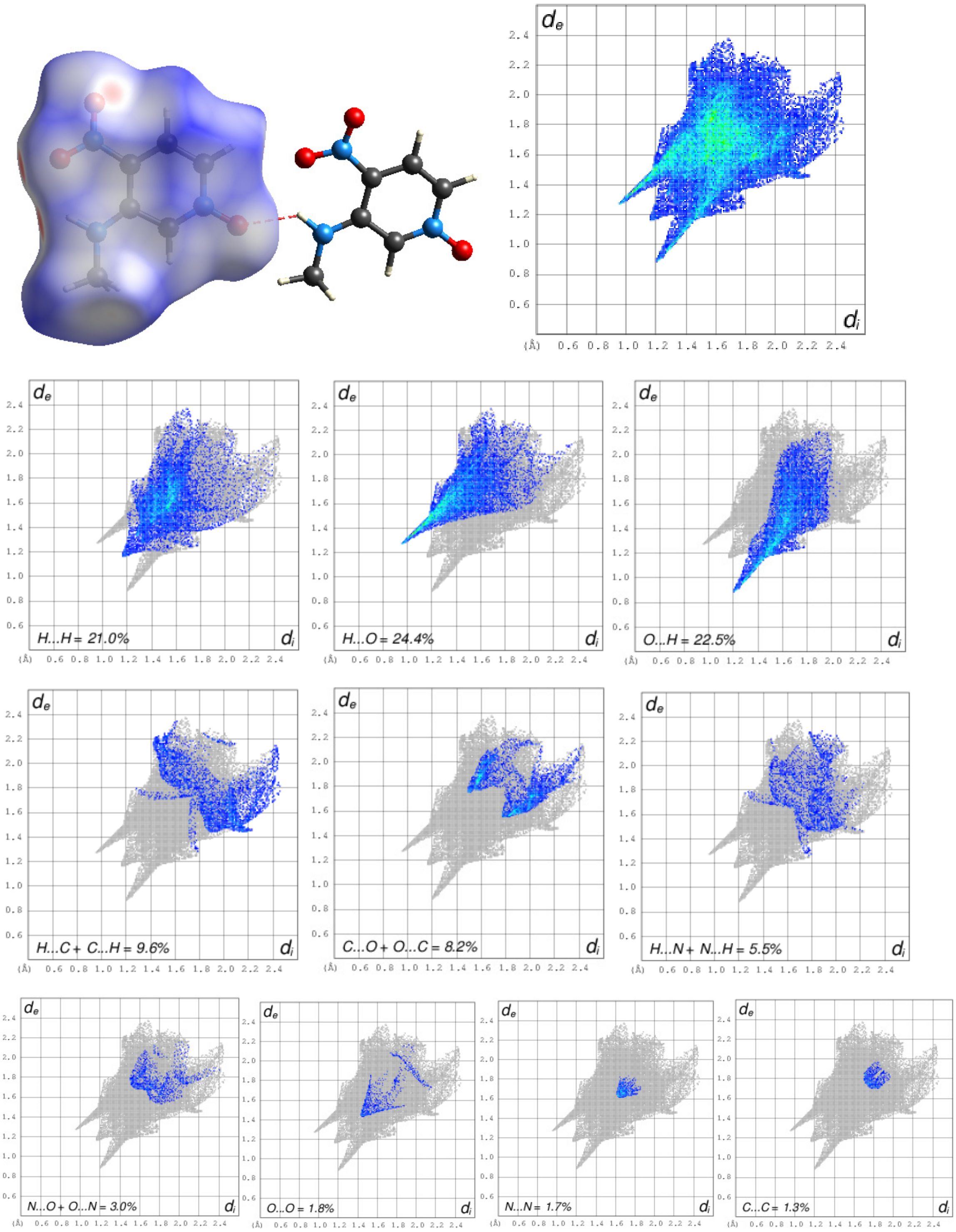

Figure S2. Deconvolution of the 2D fingerprint plots for the individual intermolecular contacts in the NOPCOOH crystal.

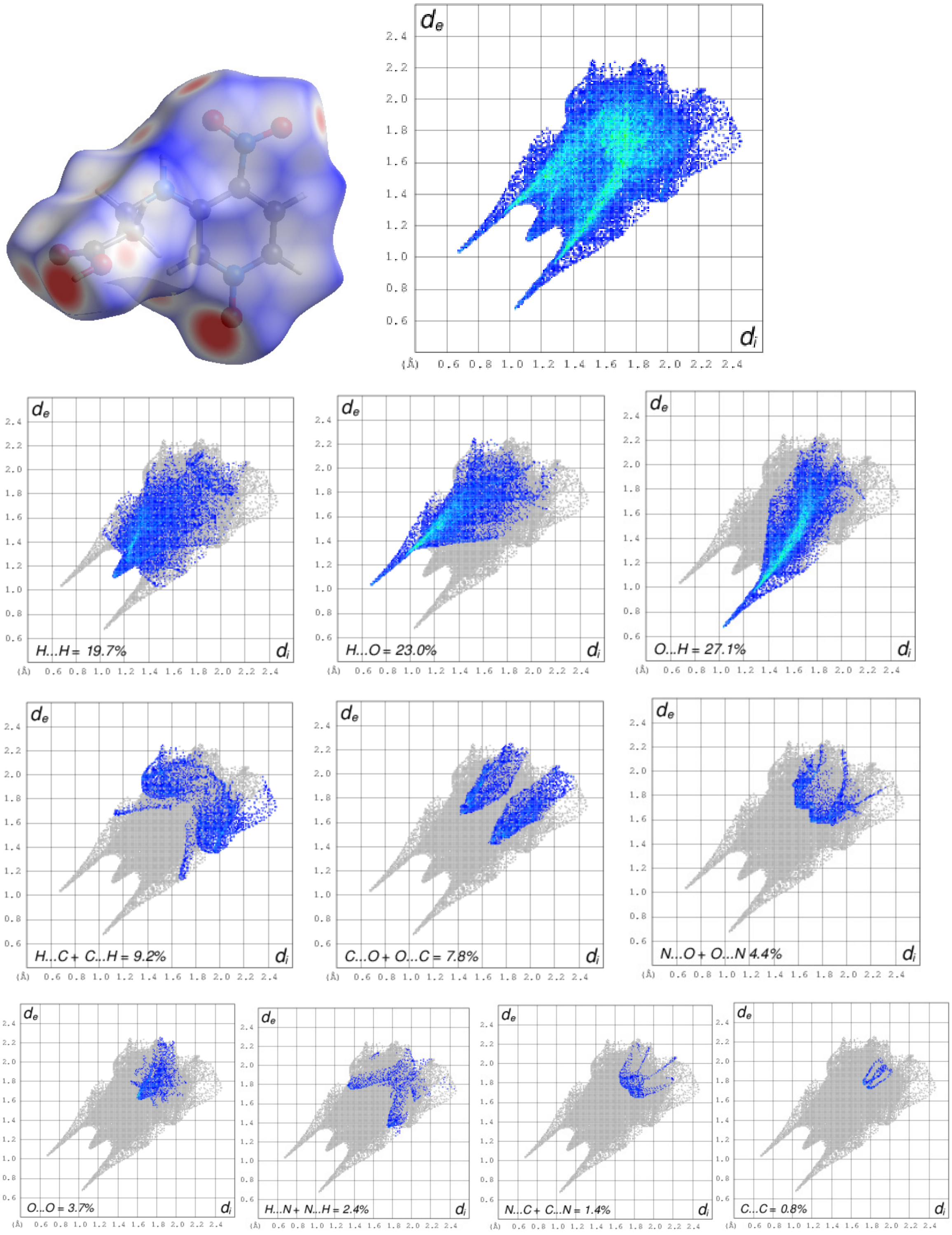

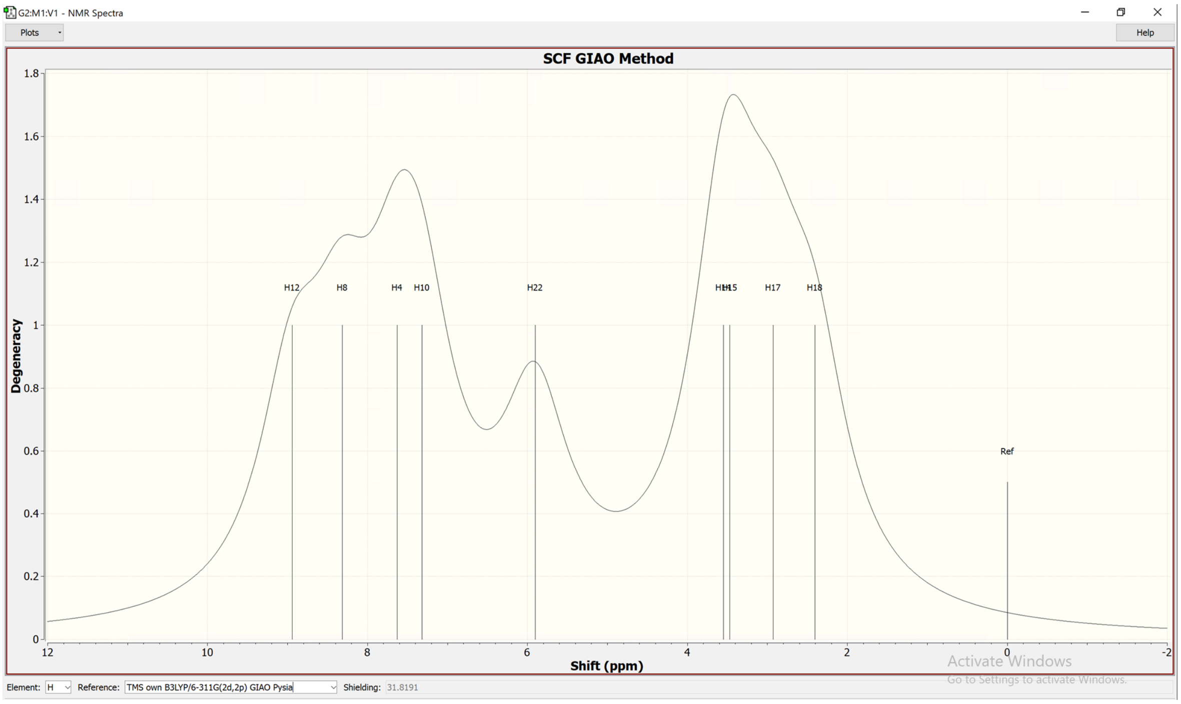

Figure S3. GIAO-calculated ^{1}H NMR spectrum of the hydrogen-bonded NOPCH$_3$ dimer model. The chemical-shift scale is shown relative to the TMS reference used in the calculation.

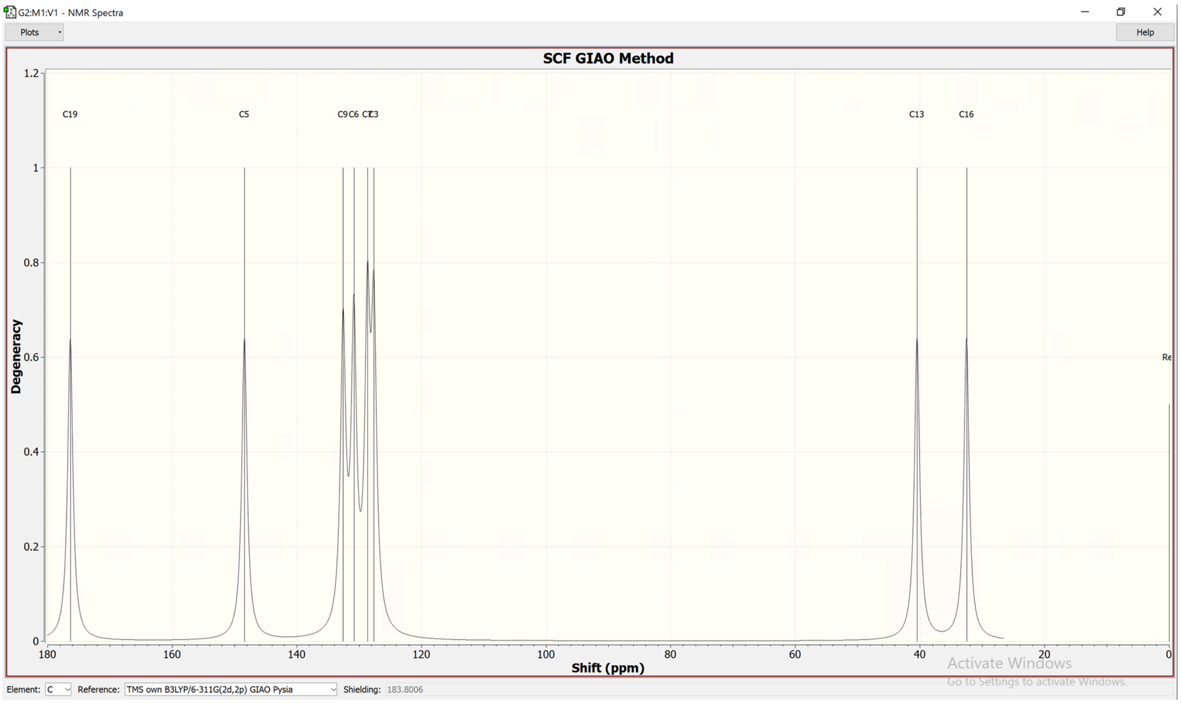

Figure S4. GIAO-calculated ^{13}C NMR spectrum of the hydrogen-bonded NOPCH$_3$ dimer model. The chemical-shift scale is shown relative to the TMS reference used in the calculation.

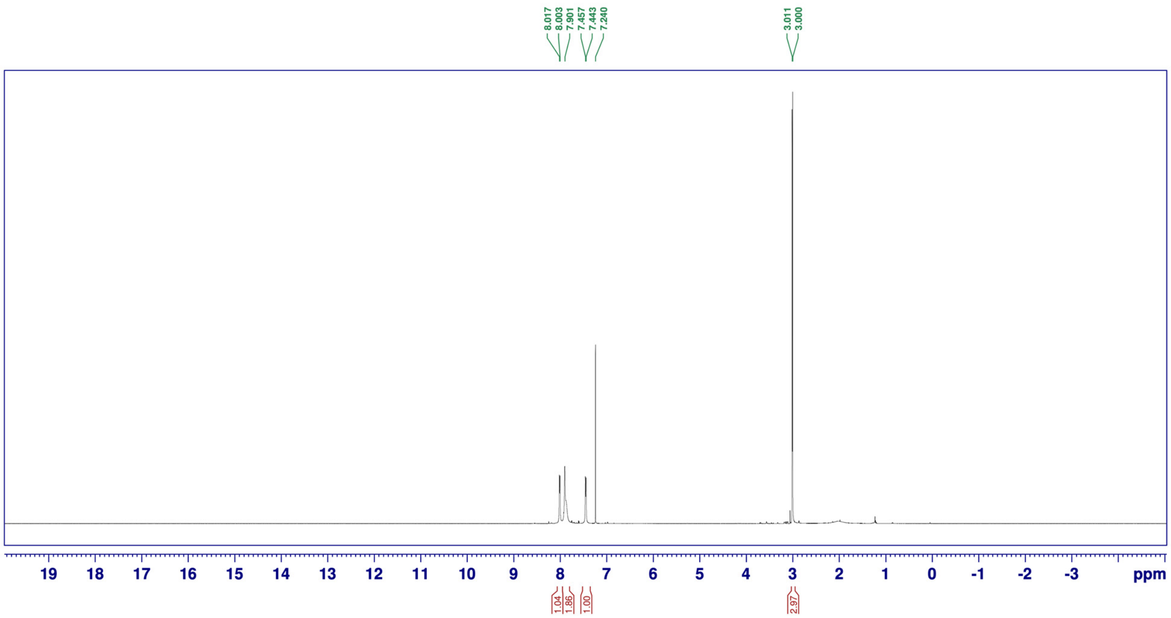

Figure S5. Solution-state ^{1}H NMR spectrum of $NOPCH_3$ recorded in $CDCl_3$.

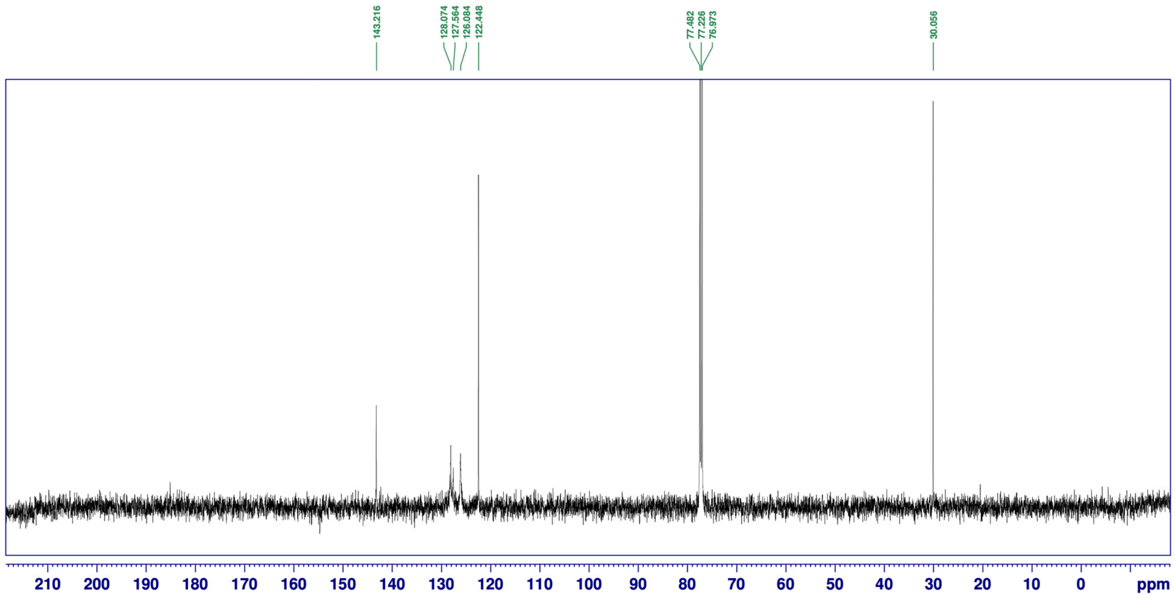

Figure S6. Solution-state ^{13}C NMR spectrum of $NOPCH_3$ recorded in $CDCl_3$.

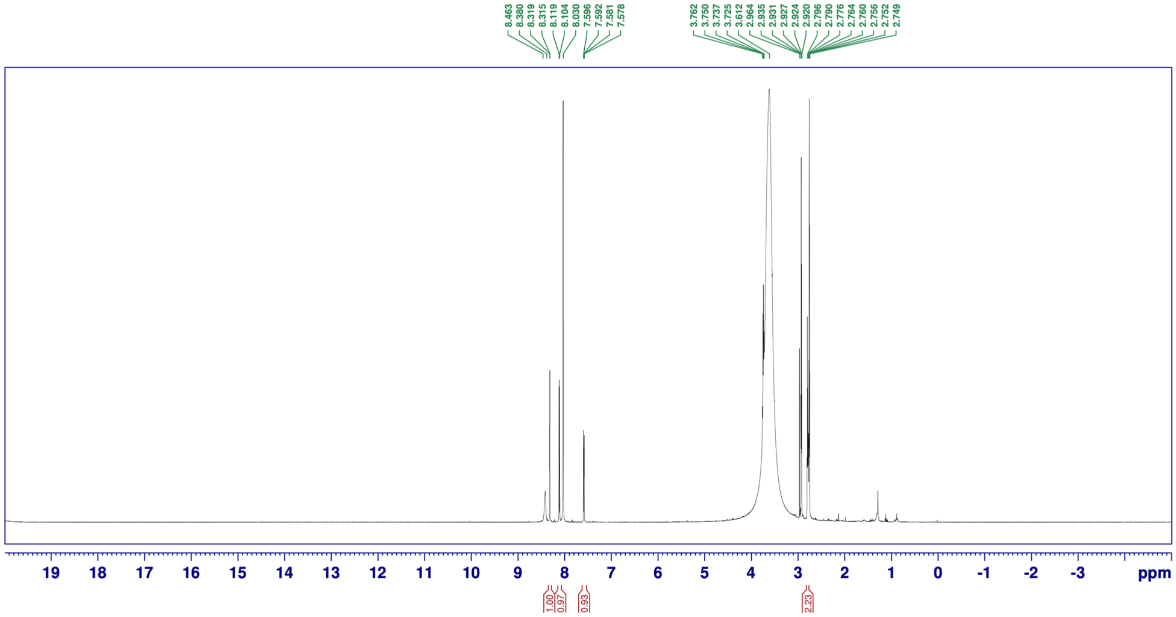

Figure S7. Solution-state ^{1}H NMR spectrum of NOPCOOH recorded in DMF-d_7.

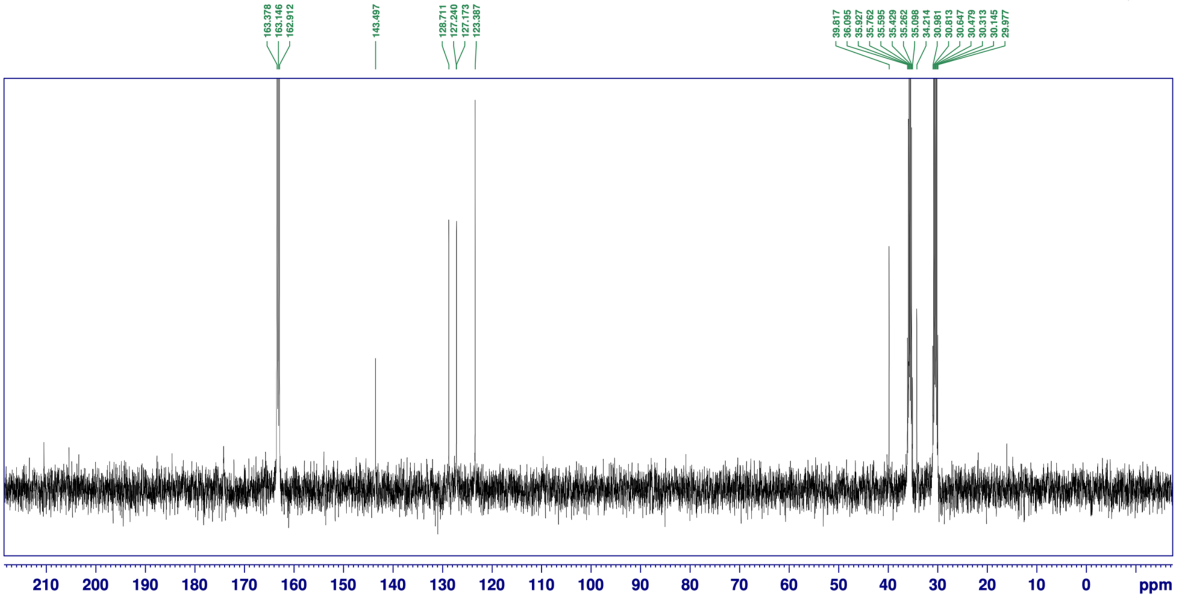

Figure S8. Solution-state ^{13}C NMR spectrum of NOPCOOH recorded in DMF-d_7.
